# Studying in vitro metabolism of the first and second generation of antisense oligonucleotides with the use of ultra-high-performance liquid chromatography coupled with quadrupole time-of-flight mass spectrometry

**DOI:** 10.1007/s00216-020-02878-0

**Published:** 2020-08-27

**Authors:** Anna Kilanowska, Łukasz Nuckowski, Sylwia Studzińska

**Affiliations:** Chair of Environmental Chemistry and Bioanalytics, Faculty of Chemistry, Nicolaus Copernicus University, 7 Gagarin Str., PL-87-100 Toruń, Poland

**Keywords:** In vitro metabolism, Human liver microsomes, Antisense oligonucleotides, Ion pair chromatography, Electrospray ionization quadrupole time-of-flight mass spectrometry

## Abstract

The aim of the present investigation was the analysis and identification of antisense oligonucleotide metabolism products after incubation with human liver microsomes regarding four different oligonucleotide modifications. Separation and detection methods based on the use of liquid chromatography coupled with quadrupole time-of-flight mass spectrometry were developed for this purpose. Firstly, the optimization of mass spectrometer parameters was done to select those which ensure the highest possible sensitivity of oligonucleotide analysis. This step was conducted for two chromatographic modes—ion pair chromatography and hydrophilic interaction liquid chromatography—due to their common application in oligonucleotide analysis. Based on sensitivity results, ion pair chromatography coupled with mass spectrometry was selected for the separation of model oligonucleotide mixtures in order to verify its selectivity for N-deleted metabolite separation. Next, the developed method was applied in the examination of oligonucleotides in vitro metabolism. First, wide optimization of incubation parameters was conducted including the concentration of the reaction buffer components. Obtained results indicated that both 3′-exonucleases and 5′-exonucleases contributed to the biotransformation of oligonucleotides. Moreover, it may be concluded that the number of metabolites depends on oligonucleotide modification and consequently its resistance to enzymatic attack. Thus, the number of the oligonucleotide metabolites decreased with the decrease of the resultant polarity of oligonucleotide caused by chemical modification.

**Electronic supplementary material:**

The online version of this article (10.1007/s00216-020-02878-0) contains supplementary material, which is available to authorized users.

**Figure:**
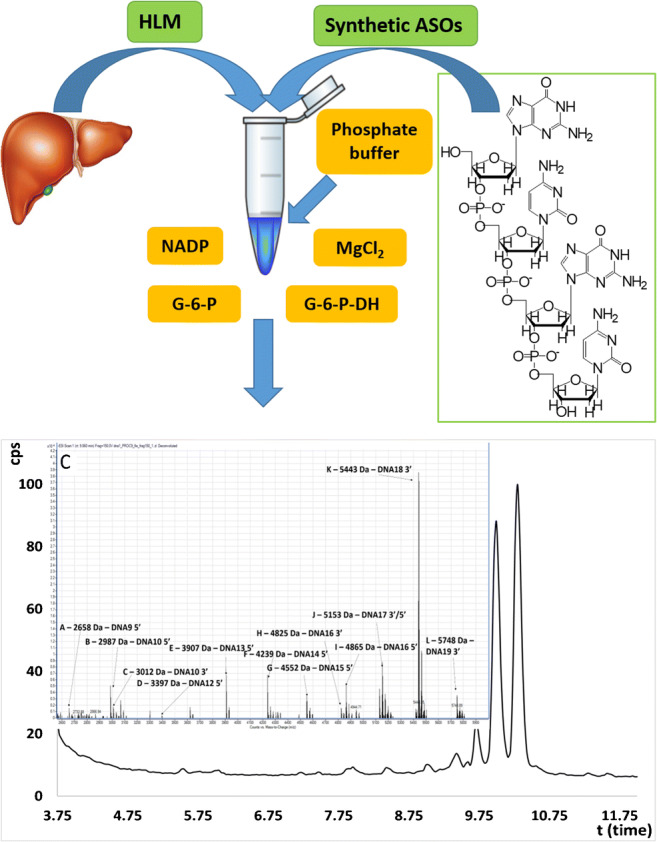
Graphical abstract

## Introduction

Antisense oligonucleotides (ASOs) are single-stranded DNA or RNA fragments, which are chemically modified by increasing their hydrophobicity and stability in the human body [1]. Modifications of ASO structure include the introduction of different groups in the polynucleotide chain such as sulfur, fluorine, methyl, methoxy, methoxyethyl groups, and methylene bridge between carbon and oxygen atoms in the pentose moieties [2]. Their therapeutic potential has been discovered in 1978 by Stephenson and Zamecnik, who found that introducing exogenous complementary oligonucleotide fragments to the virus mRNA may result in gene silencing [3, 4]. Considering that more than 40 years have passed, only nine antisense drugs, which are approved by the Food and Drug Administration, are available on the market [5, 6]. This is probably a result of certain limitations related to the characteristic structure of ASOs, which do not fulfill the so-called “rule of five” proposed by Lipiński [7, 8]. Sensitive analysis of ASOs is required for the following reasons: the short half-life of ASO in plasma, their limited stability to intracellular enzymes as well as the low concentration of metabolites in cells [7]. Identification of ASO metabolites and study of their stability and potential toxicity is especially important in terms of clinical trials, where potential drugs need to fulfill specific safety standards. Tissue homogenates and subcellular fractions are the most frequently used in vitro models for ASO metabolism studies [9, 10]. Human liver microsomes are the most frequently used in vitro model, since they are relatively cheap and rich source of enzymes responsible for drug metabolism, e.g., CYP450 and flavine-containing monooxygenases (FMO), which are first phase enzymes of biotransformation, as well as uridine glucuronide transferases (UGT) (second phase enzyme) [10].

Ion pair reversed-phase liquid chromatography (IPC) coupled with mass spectrometry (MS) as well as capillary gel electrophoresis (CGE) are common techniques, used in the in vitro as well in vivo metabolism of oligonucleotides [2, 11–13]. These analytical tools are useful in the study of ASO metabolism, the kinetics of metabolite formation, and their potential toxicity. Crooke et al. [14] investigated phosphorothioate oligonucleotide metabolism in rat liver homogenates by CGE and concluded that 3′-exonuclease, which causes enzymatic cleavage of oligonucleotide chain, had the greatest contribution to the ASOs biotransformation. However, they also observed 5′-exo- and endonuclease activity. The same conclusions were drawn by Kim et al. [9] as well as Gilar et al. [15], who studied phosphodiester and phosphorothioate oligonucleotides in biological fluids and 3′-exonuclease solution with the use of CGE technique. They observed the same metabolism products in both matrices, which confirmed the mechanism of ASO biotransformation. IPC MS has been applied by Dai et al. [12] for the phosphorothioate ASO and its metabolite identification and quantification. The developed method allowed them to identify five metabolites in the plasma and urine obtained from patients and rats. Different in vitro model was applied by Wei et al. [16], who used human liver microsomes for enzyme kinetic studies of phosphorothioate ASOs. The study performed in our group aimed also in vitro metabolism of ASOs with the use of human liver microsomes [11]. We confirmed the main ASO metabolism pathway and have shown that with the elongation of the polynucleotide chain, the resistance of ASOs to enzymatic cleavage increases.

As it has been demonstrated above, there are dozens of the reports regarding in vitro metabolism studies of ASOs with the use of different in vitro models. However, to the best of our knowledge, there are no reports concerning simultaneous studies of in vitro metabolism of several different generations of ASO with the use of human liver microsomes. For this reason, we performed such types of investigations for five oligonucleotides with different modifications and sequence lengths in order to determine the impact of the different ASOs modification on their metabolism. Moreover, wide optimization of MS parameters for two different chromatographic (IPC and HILIC) modes was performed with the use of a central composite design. This step of the study was done in order to select a technique, which could provide the greatest sensitivity of ASO analysis. It is worth mentioning that such complex optimization of MS parameters for two different chromatographic modes was performed for the first time. The optimized method was successfully applied for the separation and identification of the metabolites created during incubation with human liver microsomes.

## Materials and methods

### Materials

Unmodified and phosphorothioate oligonucleotides were obtained from Sigma-Aldrich (Gillingham, Dorset, UK), while ASOs with modified pentose (2′-*O*-methyl and 2′-*O*-methoxyethyl modifications) were purchased from Eurogentec (Seraing, Liege, Belgium). Standard 0.1 mM ASO solutions were prepared by the addition of deionized water to lyophilized form. The sequences of tested oligonucleotides, their molecular masses, and modification types are presented in Table 1.

**Table 1:** Sequences, molecular masses and modification types of studied oligonucleotides

Name	Sequence 5′→3′	Molecular weight (g/mol)	Backbone or sugar moiety modification	Mixture
DNA20	*GCCCAAGCTGGCATCCGTCA*	*6063*	*Unmodified*	*MIX1*
DNA19	GCCCAAGCTGGCATCCGTC	5750	Unmodified	
DNA18	GCCCAAGCTGGCATCCGT	5461	Unmodified	
PS20	*GCCCAAGCTGGCATCCGTCA*	*6368*	*Phosphorothioate*	*MIX2*
PS19	GCCCAAGCTGGCATCCGTC	6039	Phosphorothioate	
PS18	GCCCAAGCTGGCATCCGT	5734	Phosphorothioate	
ME20	*GCCCAAGCTGGCATCCGTCA*	*6621.5*	*2′-O-methyl*	*MIX3*
ME19	GCCCAAGCTGGCATCCGTC	6278.2	2′-*O*-methyl	
ME18	GCCCAAGCTGGCATCCGT	5959	2′-*O*-methyl	
MOE20	*GCCCAAGCTGGCATCCGTCA*	*7657*	*2′-O-methoxyethyl*	*MIX4*
MOE19	GCCCAAGCTGGCATCCGTC	7269.7	2′-*O*-methoxyethyl	
MOE18	GCCCAAGCTGGCATCCGT	6892.4	2′-*O*-methoxyethyl	
LNA11	*GCCCAAGCTGG*	*3706.4*	*Locked nucleic acid*	*MIX5*
LNA10	GCCCAAGCTG	3349.2	Locked nucleic acid	
LNA9	GCCCAAGCT	2992	Locked nucleic acid	

^a^Oligonucleotides marked in italics were used for the incubation with HLM enzymes

Mobile phases were prepared using high purity solvents such as methanol, 1,1,1,3,3,3-hexafluoro-2-propanol (HFIP) (Sigma-Aldrich, Gillingham, Dorset, UK), acetonitrile (J. T. Baker, Deventer, Holland) as well as *N*,*N*-dimethylbutylamine (DMBA), ammonium formate (Sigma-Aldrich), and LC-MS water (Merck KGaA, Darmstadt, Germany). Ammonium formate was prepared by adjusting pH to 7.5 with the use of 1% ammonia solution (Sigma-Aldrich).

The following reagents were used during the in vitro studies: nicotinamide adenine dinucleotide phosphate (NADP), glucose-6-phosphate dehydrogenase (G-6-P-DH), glucose-6-phosphate (G-6-P), sodium phosphate (Na_2_HPO_4_), sodium dihydrogen phosphate dodecahydrate (NaH_2_PO_4_·12H_2_O), microsomes derived from human liver cells (HLM) at a concentration of 10 mg/vial (Sigma-Aldrich, Gillingham, Dorset, UK). The mixture of phenol/chloroform/isoamyl alcohol (25:24:1) (VWR International, Gdańsk, Poland) was also applied.

### Instrumentation

The 1260 Infinity Quaternary System (Agilent, Waldbronn, Germany) ultra-high-performance liquid chromatography (UHPLC) system with a binary pump, vacuum-chambered microdegasser, thermostatically controlled autosampler, column compartment, and a diode array detector (DAD) was used in the present study. The system was equipped with Agilent 6540 UHD Accurate-Mass Quadrupole Time-of-Flight (Q-TOF) mass spectrometer (Waldbronn, Germany). Electrospray ionization was used in the negative ion mode for the analysis of ASOs. Full-scan mass spectra were recorded within the mass range of *m*/*z* 600–1800. The data were collected with the use of the Agilent Mass Hunter Software (version B.04.01).

Heating/cooling dry block model PCH-1 (Grants Instrument LTD, Cambs, England) and Centrifuge 5424 (Eppendorf, Hamburg, Germany) were applied during the sample preparation.

### MS parameter optimization

Optimization of Q-TOF-MS parameters was performed for two modes of liquid chromatography: IPC and HILIC. All the Q-TOF-MS parameters were optimized with the use of the central composite design (CCD) methodology, which was described in our previous papers [17, 18]. Optimization was performed for following MS parameters: drying gas flow (DGF), shielding gas flow (SGF), nebulizer gas pressure (NGP), skimmer voltage (SV), octopole voltage (OV), capillary voltage (CV), drying gas temperature (DGT), shielding gas temperature (SGT), and fragmentor voltage (FV). Experiments were carried out without chromatographic columns. In the case of IPC mode, the mobile phase consisted of 80% v/v 5 mM DMBA/150 mM HFIP and 20% v/v of methanol. The mobile phase composition for HILIC mode was as follows: 30% v/v of 5 mM ammonium formate (pH 7.5) and 70% v/v of acetonitrile. The injection volume equaled 0.5 μL.

After optimization, both chromatographic modes were compared regarding ASO EIC peak areas. This step of the study was also performed without the chromatographic column for all tested oligonucleotides and their N-1 and N-2 synthetic metabolites (Table 1) at concentration 25 μM and injection volume 0.5 μL.

### Chromatographic conditions

Oligonucleotide separation and in vitro metabolism studies were performed in IPC mode with the use of ACE Excel 1.7 C18 column (1.7 μm; 2.1 × 100 mm; Advanced Chromatography Technologies, Aberdeen, Scotland). The mobile phase flow rate was in range the 0.3–0.35 mL/min. The autosampler temperature was 30 °C, while the column was kept at 50 °C. The injection volume was 3 μL. Firstly, chromatographic methods were developed for the separation of five different model ASO mixtures (parent compounds and their N-1 and N-2 synthetic metabolites) with the use of gradient elution. Table S1 in the Electronic Supplementary Material (ESM) presents optimized gradient elution programs, which were used for the separation of model ASO mixtures. However, during the analysis of the samples obtained from in vitro metabolism studies developed chromatographic methods were modified due to the matrix effect. More detailed information regarding this step of the study was described in the “Full scan mass spectra of ASOs” section.

### Incubation of ASOs with HLM enzymes

Each incubation of ASOs with HLM was done in 100 mM phosphate buffer (pH 7.4) with the use of the NADPH regenerating system composed of NADP, G-6-P, G-6-P-DH, and MgCl_2_ at various concentrations. After 30-min preincubation of ASOs with reaction buffer (without NADP) at 37 °C, the reaction was initiated by the addition of NADP at total sample volume 200 μL and incubated for 12 h. ASO concentration equaled 20 μM. Optimization of the incubation conditions was performed during the study, including concentration changes of the HLM and NADPH regenerating system components. The blank samples were prepared in the same way as samples with ASOs; however, oligonucleotides were replaced by appropriate volume of phosphate buffer. The process was quenched with the addition of 200 μL of phenol/chloroform/isoamyl alcohol. Samples after incubation were prepared with the use of a liquid–liquid extraction (LLE) methodology (described in the “Sample preparation” section) and then analyzed by UHPLC Q-TOF-MS. Each assay was prepared in two repeats.

The following final incubation parameters were selected for the study of ASOs in vitro metabolism: 20 μM of ASO, 2 mg/mL of HLM, 10 mM of NADP, 0.6 U/mL of G-6-P-DH, 10 mM of G-6-P, and 4.5 mM of MgCl_2_.

### Sample preparation

LLE technique with the use of phenol/chloroform/isoamyl alcohol mixture (25:24:1 v/v) was applied during present investigations. The procedure was performed according to the literature [18–20]. Based on these data, this procedure provided recovery of approximately 80% for ASOs. In our study, the recovery and validation parameters were not determined due to the application of the developed method only for qualitative investigation. Two hundred microliters of phenol/chloroform/isoamyl alcohol mixture was added to the 200 μL of the sample after incubation and mixed. Next, the suspension was centrifuged (RCF = 20,160×*g* for 35 min). About 160 μL of supernatant was transferred to another tube and then an additional LLE extraction step was made with the use of chloroform. Such extraction was repeated five times to remove phenol residues.

## Results and discussion

### Optimization of Q-TOF-MS parameters with the use of CCD in IPC and HILIC mode

In order to develop a sensitive method of ASO analysis, the optimization of Q-TOF-MS parameters has been done. ESI-Q-TOF-MS was selected for the ASO metabolism studies because it enables high accuracy mass measurement, which is particularly important for qualitative analysis. Optimization was performed for four selected oligonucleotides (DNA20, PS20, ME20, and MOE20) in two different modes of chromatography: IPC and HILIC. According to the literature, IPC mode allows for selective separation of N-deleted ASO metabolites; however, quantification levels in MS detection (LOQ > 2.5 ng/mL) need further improvement [2, 19, 21]. On the other hand, HILIC mode provided lower limits of quantification (LOQ > 0.02 ng/mL) compared to IPC; however, selective separation of shorter metabolites is difficult [22–25]. For this reason, we decided to compare the sensitivity of Q-TOF-MS for these two chromatographic modes regarding ASO TIC peak areas. The following mobile phases were selected: 5 mM DMBA/150 mM HFIP in a mixture with methanol as well as 5 mM ammonium formate at pH 7.5. In our previous investigations, these mobile phases provided the greatest possible sensitivity of ASO determination by MS detection [20, 25]. Moreover, the application of the mixture composed of DMBA and HFIP allowed us to obtain selective separation of complex ASO mixture [20], while in HILIC mode separation efficiency was limited [25].

**Fig. 1:**
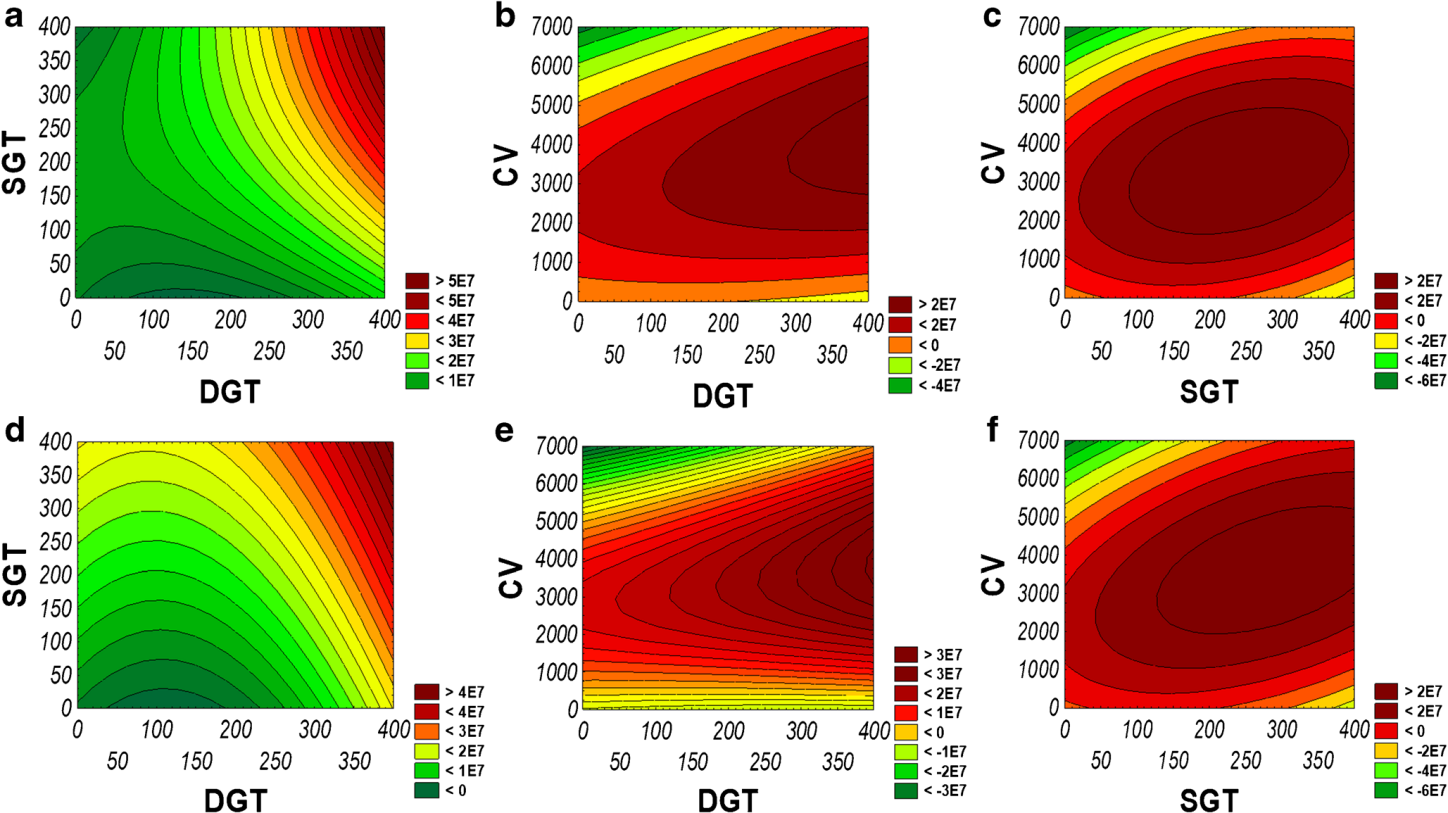
Exemplary 2D contour plots obtained plotting CCD equation for Q-TOF-MS optimization for DNA20 in IPC (**a**–**c**) and HILIC (**d**–**f**) mode: **a** SGT vs. DGT is plotted, **b** CV vs. DGT is plotted, **c** CV vs. SGT is plotted, **d** SGT vs. DGT is plotted, **e** CV vs. DGT is plotted, **f** CV vs. SGT is plotted. For each graph, the third variable is TIC peak area

MS parameters were optimized in order to obtain the highest possible sensitivity of ESI-Q-TOF-MS analysis of oligonucleotides. Similar optimization was performed for both chromatographic modes. DGF, SGF, NGP, SV, OV, CV, DGT, SGT, and FV were independent variables. These MS parameters have been divided into three sets of variables: (1) DGT (40–340 °C), SGT (43–380 °C), CV (600–6000 V); (2) SV (18–300 V), OV (17–800 V), FV (20–320 V); (3) DGF (1.8–10 L/min), SGF (8–11 L/min), and NGP (15–50 psig). The dependent variable was the ASO peak area on the total ion chromatogram (TIC). All chromatographic analyses were performed in duplicate. The range of the tested values and TIC peak areas for DNA20 are presented in the ESM (Table S2). Similar tendencies were observed for the remaining ASOs. Based on the obtained results, 2D contour plots were prepared for each of ASO. It has to be noted that similar effects were observed for each of the tested ASO and for this reason, we present results only for exemplary DNA20. Figure 1 shows the results obtained for the first set of variables obtained in both chromatographic modes. 2D contour plots for the second and third sets of variables in IPC and HILIC mode are presented in the ESM (Figs. S1 and S2). As can be seen from contour plots, regardless of the applied chromatographic mode, similar tendencies were observed for the first and second sets of variables for all tested ASOs (Fig. 1 and ESM Fig. S1). Contrary to these findings, different optimum regions of TIC area in IPC and HILIC mode were obtained in the case of the third set of variables (DGF, SGF, NGP) (ESM Fig. S2). Based on 2D contour plots for each set of variables for all tested ASOs in both chromatographic modes, optimal parameters providing the highest TIC peak areas were selected and presented in Table 2. It should be noted that the FV value was lowered to 150 V, due to the partial ASO fragmentation in ion source for its higher values.

**Table 2:** Optimal Q-TOF-MS parameters for ASO analysis selected based on the 2D contour plots

Optimal Q-TOF-MS parameters		
	IP RP HPLC	HILIC
Shielding gas temperature (SGT) (°C)	350	350
Drying gas temperature (DGT) (°C)	400	400
Capillary voltage (CV) (V)	4000	4000
Octopole voltage(OV) (V)	800	800
Fragmentor voltage (FV) (V)	350 (150^a^)	350 (150^a^)
Skimmer voltage (SV) (V)	60	60
Drying gas flow (DGF) (L/min)	11	3
Shielding gas flow (SGF) (L/min)	8	12
Nebulizer gas pressure (NGP) (psig)	50	15

^a^Fragmentor voltage was lowered to 150 V due to partial ASO fragmentation in the ESI source and for higher FV values

It should be noted that such comprehensive optimization allowed us to obtain the highest possible sensitivity for ASO analysis, which is especially important considering metabolism studies: the greater the sensitivity of the method, the greater the possibility of detection metabolites at the low level of concentrations. Optimized MS parameter conditions were applied for the comparison of IPC and HILIC mode considering MS sensitivity.

### Full scan mass spectra of ASOs

After the optimization of ESI-Q-TOF-MS parameters, full scan mass spectra for all tested oligonucleotides were recorded for both chromatographic modes in order to select precursor ions characterized by the greatest intensity. These signals were further used for comparison of EIC peak areas in IPC and HILIC mode. Exemplary mass spectra obtained for PS19 for both techniques are presented in ESM Fig. S3. The same signal has the greatest intensity for both chromatographic modes 1508.64 *m*/*z*. For the remaining oligonucleotides, analogical tendencies were observed and for this reason, following precursor ions were selected for both chromatographic techniques: DNA20–1514.51 Da, DNA19–1436.25 Da; DNA18–1363.99 Da; PS20–1590.66 Da; PS18–1432.39 Da; ME20–1654.30 Da; ME19–1568.28 Da; ME18–1448.77 Da; MOE20–1541.51 Da; MOE19–1452.76 Da; MOE18–1721.93 Da; LNA11–1234.20 Da; LNA10–1115.189 Da. Only for LNA9 different precursor ions were selected for both chromatographic modes: 996.17 Da for HILIC and 1494.72 Da for IPC. It needs to be underlined that regardless of the tested oligonucleotide, signals obtained for IPC mode are characterized by greater intensity compared to HILIC (ESM Fig. S3). Moreover, spectra obtained for HILIC mode were more complex due to the presence of the sodium adducts.

### Comparison of EIC peak areas of ASO in IPC and HILIC

In order to select liquid chromatography mode, which would provide the highest possible sensitivity of ASO analysis, IPC and HILIC were compared regarding ASOs’ EIC peak areas. EIC peak areas obtained for both chromatographic techniques are shown in Table 3.

**Table 3:** EIC peak areas of tested oligonucleotides for IPC and HILIC mode

ASOs	HILIC mode	IPC mode
	30% v/v 5 mM pH 7.5 ammonium formate and 70% v/v ACN	80% v/v 5 mM DMBA/ 150 mM HFIP and 20% v/v MeOH
	ASOs EIC peak areas	
DNA20	107,858	*274,197*
DNA19	90,785	*205,048*
DNA18	119,271	*365,343*
PS20	39,390	*160,701*
PS19	77,134	*132,705*
PS18	84,239	*115,535*
ME20	21,450	*43,834*
ME19	55,066	*72,692*
ME18	*71,180*	64,929
MOE20	*73,388*	49,893
MOE19	*148,297*	96,533
MOE18	*177,685*	138,373
LNA11	34,510	178,541
LNA10	174,103	189,509
LNA9	*161,571*	110,749

^a^Higher values for each chromatographic mode were marked in italics

Based on these results, it may be observed that greater EIC peak areas were obtained for IPC mode for most of the tested ASOs. However, opposite tendencies were obtained for all oligonucleotides modified with 2′-*O*-methoxyethyl group as well as LNA9. EIC peak areas for these compounds were about 1.5 times greater in the case of HILIC. This is probably related to the more effective ionization of these oligonucleotides in ammonium formate compared to DMBA/HFIP mixture. For unmodified oligonucleotides, more than the twofold increase of EIC peak areas was observed for IPC compared to HILIC. Such an effect was probably a result of the lower pH of the mobile phase in HILIC mode and the high content of the acetonitrile. This consequently led to the disturbing desorption of these compounds from the droplet, resulting in lower EIC peak areas. Significantly higher peak areas in IPC were also observed for phosphorothioates. In the case of LNA11 and LNA10 greater peak area was determined in IP RP HPLC mode. It may be assumed that IPC mode is a better choice for the analysis of unmodified, phosphorothioate, and 2-*O*-methyl modifications of ASOs when ESI-Q-TOF-MS is applied. However, based on our previous experience, IPC mode allowed us to obtain selective separation of the complex ASO mixture, while in the case of HILIC mode, full separation of the mixture composed of oligonucleotides was difficult to achieve [20, 25]. For this reason, IPC mode was selected for further investigations*.*

### Development of ASOs and their synthetic metabolite separation methods in IPC

**Fig. 2:**
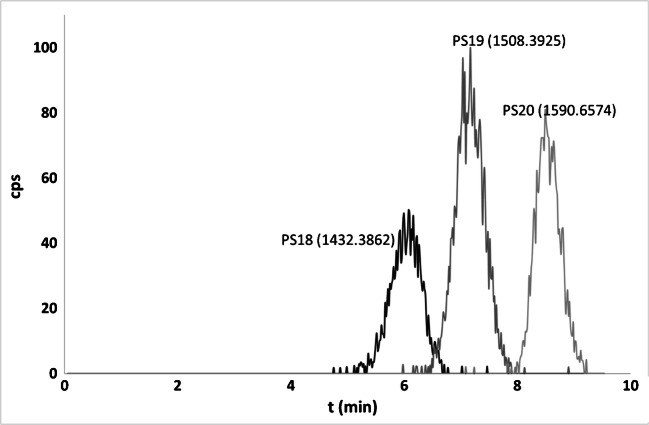
Exemplary EIC chromatogram for PS20, PS19, and PS18 mixture. Chromatographic conditions: see the Experimental section and Table S1 in the Supplementary Materials

Five ASO standard mixtures (Table 1) were selected in order to verify the selectivity of the IPC-based method, regarding the separation of N-deleted metabolites from parent compounds. Mixtures components were selected based on the main biotransformation pathway of ASOs (sequential nucleotide deletions mediated by 3′-exonucleases). This step of the study was useful in the determination of the IPC suitability for ASO in vitro studies. In order to obtain the best possible selectivity of ASO separation, some parameters were changed, such as gradient elution program, column temperature, and mobile phase flow rate. In the case of the last parameter, the limitation was the backpressure generated by the system. As can be seen in Table S1 in the ESM, different gradient elution programs were developed for each tested mixture due to the differences in the length of the sequences, as well as the resultant polarity caused by various chemical modifications of these compounds. Exemplary EIC chromatogram obtained for PS20 and its metabolites is presented in Fig. 2, while chromatograms for the remaining mixtures are shown in the ESM (Figs. S4–S7). Based on these figures, it may be observed that only for MIX1 obtained peaks are smooth (ESM Fig. S4). It is related to the oligonucleotides structure: introducing a modification to their structure increases their hydrophobicity, and consequently, ionization effectiveness of such compounds is lower.

As can be seen on these figures, the application of gradient elution mode allowed to obtain effective separation of all tested mixtures in less than 10 min. Moreover, it can be noted that the elution order of ASOs depends on the sequence length: parent compounds were eluted after their N-2 and N-1 shorter synthetic metabolites, for all the studied oligonucleotides. In summary, UHPLC in ion pair mode is an effective tool for the analysis of ASO metabolite mixtures, because this technique is characterized by acceptable selectivity of separation, short analysis time, and reduction of analytes and solvent consumption.

It has to be underlined that considering the analysis of ASO samples obtained after incubation with HLM, we had to change gradient elution programs and mobile phase flow rates for all tested oligonucleotides. This was due to the fact that the applied sample preparation method (LLE) allowed us to purify samples from proteins; however, it did not remove NADP, which was present in the samples at high concentration. This compound (eluted in the void time for the developed gradient programs) caused a significant signal suppression and shift of analyte retention. For this reason, developed chromatographic methods were modified by increasing the percentage of MeOH in the mobile phase, and consequently, the mobile phase flow rate had to be reduced due to the limited operating pressure capability of the instrument. In order to reduce signal attenuation, fraction from the column was switched to the mass spectrometer at 3.75 min after injection. Consequently, NADP was not loaded to MS. Gradient elution programs applied during ASO sample analysis after incubation are also presented in Table S1 in the ESM.

### Optimization of ASOs in vitro incubation conditions with the use of HLM enzymes

HLM were selected for ASO incubation. Five different oligonucleotides, differing in modification type were used for this purpose, including DNA20, PS20, ME20, MOE20, and LNA11 (Table 1).

In order to select incubation parameters, which could provide the greatest number of the ASO metabolites, different values of these parameters concentrations were tested, including HLM (concentration ranging from 0.8 to 3 mg/mL), NADP (1.3–20 mM), G-6-P-DH (0.4–3 U/mL), 6-G-P (concentration between 6.6 and 35 mM), and magnesium ions (3.3–20 mM). The starting incubation conditions have been selected based on product information provided by the manufacturer and these were 0.8 mg/mL HLM, 1.3 mM NADP, 0.4 U/mL G-6-P-DH, 6.6 mM G-6-P, and 3.3 mM MgCl_2_. All incubation procedures were performed in the phosphate buffer medium (0.1 M at pH = 7.4) during 12 h and 20 μM of each ASO. Table 4 presents all procedures applied during the optimization of incubation parameters.

**Table 4:** Incubation procedures applied during the study of ASO in vitro metabolism with the use of human liver microsomes

Procedure	1	2	3	4	5	6	7	8	9	10	11	12	13	14	15
ASOs (μM)	20	20	20	20	20	20	20	20	20	20	20	20	20	20	20
HLM (mg/mL)	0.8	0.8	0.8	1.6	1.6	2	3	2	2	2	2	2	2	2	2
NADP (mM)	1.3	2.6	6.5	6.5	10	10	20	10	10	10	10	10	10	10	10
G-6-P-DH (U/mL)	0.4	0.4	0.4	0.4	0.4	0.4	0.4	0.4	0.6	1	1	2	3	1	1
G-6-P (mM)	6.6	6.6	6.6	6.6	6.6	6.6	6.6	10	10	15	15	25	35	10	15
MgCl_2_ (mM)	3.3	3.3	3.3	3.3	3.3	3.3	3.3	3.3	4.5	10	15	15	20	3.3	3.3

#### The influence of the NADP and HLM concentration

By increasing NADP concentration in the range of 1.3–6.5 mM for procedures 1–3 caused an increase in the number of formed metabolites for DNA20, PS20, and LNA11 (Table S3 in the ESM; Table 4). N-1, N-2, and N-3 shorter metabolites formed by enzymatic cleavage from 3′ and 5′ ends of DNA20 were detected for procedures 1 and 2 (1.3 mM and 2.6 mM of NADP), while by increasing of NADP concentration to 6.5 mM (procedure 3; Table 4) resulted in the formation of one additional metabolite (N-4 shorter at 5′ end) (ESM Table S3). Considering PS20, it may be observed that the increase of NADP concentration from 1.3 mM (procedure 1) to 2.3 mM (procedure 2) resulted in the creation of 5′N-1 and 3′N-2 metabolites for PS20. The N-1 metabolites from 3′ and 5′ ends, as well as hydrated 5′N-2 metabolites were identified for LNA11 (ESM Table S3; procedure 2). Further increasing of NADP concentration to 6.5 mM (procedure 3 in Table 4 and ESM Table S3) caused the creation of 5′N-2 metabolite for PS20 and 3′N-3 shorter metabolism product for LNA11 (ESM Table S3). These results confirmed that the main ASOs’ metabolic pathway is based on the cleavage of nucleotides, which are present at the end of the polynucleotide chain, by both 3′-exonuclease and 5′-exonuclease. Based on results presented in ESM Table S3, it may be observed that despite the increasing of NADP concentration, no metabolites were observed for ME20 and MOE20, which are characterized by the high resistance for the enzymatic attack [2, 9, 17].

With the increase of the NADP concentration from 6.5 mM (procedure 4) to 10 mM (procedure 6) together with HLM concentration (from 0.8 to 2 mg/mL) resulted in the further formation of chain-shortened metabolites of DNA20, including 5′N-5, 5′N-7, and 3′N-10 metabolism products (ESM Table S3, procedures 4–6). Similar tendencies were observed for ME20 and four N-1 and N-2 shorter metabolites were identified (ESM Table S3, procedures 4–6). However, further increasing of HLM to 3 mg/mL (procedure 7 in Table 4) did not affect the number of metabolites for ME20, while in the case of PS20, one more metabolite was detected for higher HLM concentration compared to the lower one (ESM Table S3, procedures 6 and 7).

In the case of LNA, when 6.5 mM NADP and 0.8 mg/L HLM concentrations were applied (procedure 4 in Table 4 and ESM Table S3), metabolites formed by cleavage of one, two, and three nucleotides from 3′ and 5′ ends were detected. Further increasing NADP and HLM concentrations (ESM Table S3 and Table 4, procedure 7) led to the creation of additional metabolites, which were shorter by four and five nucleotides compared to parent ASO.

Simultaneous increase of HLM and NADP concentrations (procedures 4–7, ESM Table S3) for PS20 did not provide a greater number of metabolites compared to procedure 4. Four metabolites were formed for procedure 4 as well as procedure 7, but they were differing by length, depending on applied procedure: 5′N-1, N-4 from 3′ and 5′ ends and 5′N-5 shorter oligonucleotides for procedure 4, N-1 from 3′ and 5′ ends, 5′N-5 and 5′N-6 shorter compounds for procedure 7 (Table 4 and ESM Table S3). In the case of MOE20, by increasing the HLM together with NADP concentration did not affect the enzymatic reaction, and consequently, for procedure 7, no metabolites were formed (ESM Table S3).

#### The influence of the G-6-P concentration

Another factor that has a great impact on the metabolism of tested oligonucleotides is G-6-P concentration. Obtained results indicated that the greatest number of the ASO metabolites created during incubation was observed only in the concentration range between 6.6 and 10 mM (Table 4 and ESM Table S3, procedures 6–8). Such tendency was especially apparent for DNA20 and PS20, for which the increase of G-6-P concentration from 6.6 mM (procedure 6) to 10 mM (procedure 8) resulted in the production of five and nine additional metabolites for DNA20 and PS20 respectively (ESM Table S3, procedure 8). For DNA20, both 3′ end and 5′ end metabolism products were identified for procedure 8, including 3′N-1, 3′N-3, 3′N-4, 3′N-10, and 3′N-11 as well as 5′N-1–5′N-8 and 5′N-11 chain-shortened metabolites (ESM Table S3, procedure 8). Considering PS20 for procedure 8, N-1–N-6 shortened oligonucleotides from 3′ end were observed, while N-1–N-8 metabolites from 5′ end (ESM Table S3).

For LNA modification eight N-1–N-5 shorter metabolites were identified for procedures 6 and 8 (ESM Table S3) and their number was the same regardless of the procedure used. Therefore, it may be concluded that G-6-P concentration did not influence LNA11 enzymatic degradation.

In the case of ME20, by increasing of G-6-P concentration from 6.6 to 10 mM for procedures 6–8 (Table 3 and ESM Table S3) resulted in the identification of further products of N-deletion. Besides 3′N-1 and 5′N-1 and 3′N-2 metabolism products, which were detected for procedure 6, also additional metabolite (5′N-2) was observed for procedure 8 (ESM Table S3). Similarly, as in the case of increasing of NADP and HLM concentration, increasing of G-6-P concentration did not yield satisfactory results in the case 2′-O-methoxyethyl-modified ASO and no metabolites of MOE20 were identified for procedure 8 (ESM Table S3).

It is important to notice that further increasing of G-6-P concentration in the range between 10 and 35 mM (for procedures 10–13, Table 4 and ESM Table S3) not yielded satisfactory results—similar or even lower number of metabolites were formed for all tested oligonucleotides (ESM Table S3).

#### The impact of the G-6-P-DH concentration

The effect of the G-6-P-DH concentration on the ASO biotransformation was also examined. By increasing its concentration in the range of 0.4–0.6 U/mL for procedures 8 and 9 (Table 4) resulted in an increment of the metabolites number: for DNA20, 15 metabolites were identified for 0.6 U/mL (ESM Table S3, procedure 9). It has to be mentioned that some metabolites were different, depending on the procedure number. 5′N-11 metabolite was identified only for the lower G-6-P-DH concentration (ESM Table S3, procedure 8), while 5′N-9 metabolite was observed only for the higher concentration of this parameter (ESM Table S3, procedure 9). A similar tendency was noted for PS20 and LNA11: with the increase of G-6-P-DH concentration, one more metabolite was identified compared with the lower G-6-P-DH concentration (procedures 8–9, Table 4).

Considering ME20, the number of metabolites was not changed for procedures 8 and 9 and 5′N-1, 5′N-2, 3′N-1, and 3′N-2 metabolites were observed. By increasing of G-6-P-DH concentration for MOE20 caused the production of only one metabolite, which was 3′N-1 (ESM Table S3, procedure 9). This indicates the high resistance of MOE20 for enzymatic cleavage.

It has to be emphasized that the impact of G-6-P-DH and G-6-P concentrations is closely related and the greatest number of metabolism products for each ASO was observed only for the following range of concentrations: 6.6–10 mM G-6-P and 0.4–0.6 U/mL G-6-P-DH (procedures 8 and 9, ESM Table S3). Further increasing of these concentrations resulted in a decrease of metabolites number (procedures 10–13, ESM Table S3). This is probably related to the fact that both of them are responsible for the reduction of NADP^+^ to NADPH, which is then oxidized by CYP enzymes to NADP and consequently the enzymatic reaction is prolonged. When the concentration of these parameters is too high, the equilibrium of the NADPH regeneration reaction is probably disturbed, leading to a reduction in the number of produced metabolites (procedures 10–13, ESM Table S3).

#### Effect of MgCl_2_ concentration

The last important parameter, which influenced oligonucleotide metabolism was the concentration of magnesium ions, which are the G-6-P-DH cofactor. By increasing their concentration contributed to the increase of the metabolites number, however, only in the range of concentration between 3.3 and 4.5 mM (ESM Table S3, procedures 8 and 9). For the 4.5 mM of MgCl_2_ (procedure 9, ESM Table S3), one additional metabolite was created for all tested ASOs, compared to the 3.3 mM of MgCl_2_ (procedure 8, ESM Table S3), except ME20 for which the number of metabolism products has not changed (ESM Table S3, procedure 9). Following additional metabolites were identified for tested compounds: 5′N-11 for DNA20, 3′N-2 for PS20, 3′N-5 for LNA11, and 3′N-1 for MOE20 (ESM Table S3, procedure 9). Further increasing magnesium ion concentration to 35 mM led to decreasing the number of metabolites (ESM Table S3, procedures 10–13). It was particularly apparent for modified ASOs, where reduction of metabolites was significant—no metabolites of PS20, ME20, and MOE20 were detected for 35 mM of MgCl_2_ (ESM Table S3, procedure 13), while only 3′N-2 and 3′N-3 metabolites were identified for LNA11 (ESM Table S3, procedure 13). This is probably related to the fact that this parameter is a cofactor of G-6-P-DH and the simultaneous increase of concentrations of these parameters resulted in the destabilization of the NADPH regeneration, and consequently, the enzymatic reaction was not as efficient as for the lower concentrations of G-6-P-DH and MgCl_2_.

#### Comparison of different ASO modifications on their biotransformation

Based on the obtained results, it may be observed that the greatest number of metabolites was created for the unmodified oligonucleotide. For the remaining ASOs, the number of metabolites decreased in the following order: PS > LNA > ME > MOE. Such tendencies are the result of their structures and consequently different resistance for enzymatic digestion.

### Identification of metabolites

ASOs do not undergo P450 oxidative metabolism and their main metabolic pathway in vitro as well as in vivo is their sequential nucleotide deletion by 3′-exonucleases and 5′-endonucleases [15, 16, 26–28]. However, Kim et al. [9] in the recent paper identified some oxidation products of PS ASO backbone present in the mouse lung samples after inhalation administration of Eluforsen (PS ASOs with 2′-O-ME modifications).

The dynamics of nuclease-mediated biotransformation process is determined by the type of modification in the ASO structure. In vivo, unmodified oligonucleotides are rapidly degraded and excreted with a few minutes plasma half-life, while the introduction of phosphorothioate groups into ASO backbone or sugar modifications at 2′ position in the ribose structure increases their resistance against nucleases, whereby they are able to reach target tissues and cells [26, 29–32]. As a consequence, the plasma half-life of these compounds is extended from several hours to even months [12, 15, 33–37]. It should be noted that for some ASOs, endonucleases may also contribute to their metabolism. This mainly concerns compounds with modified sugar moieties at 3′ and 5′ ends, which consequently results in their greater resistance against exonucleases and initial exonuclease cleavage within the central ASOs region. Further degradation of exposed deoxynucleoside ends mediated by exonucleases also occurs [28, 30, 35, 38, 39]. Concerning ASOs modified within each sugar moieties, it was reported that metabolism of such compounds was also mediated by 3′- and 5′-exonucleases; however, their biotransformation is significantly more slowly [9].

**Fig. 3:**
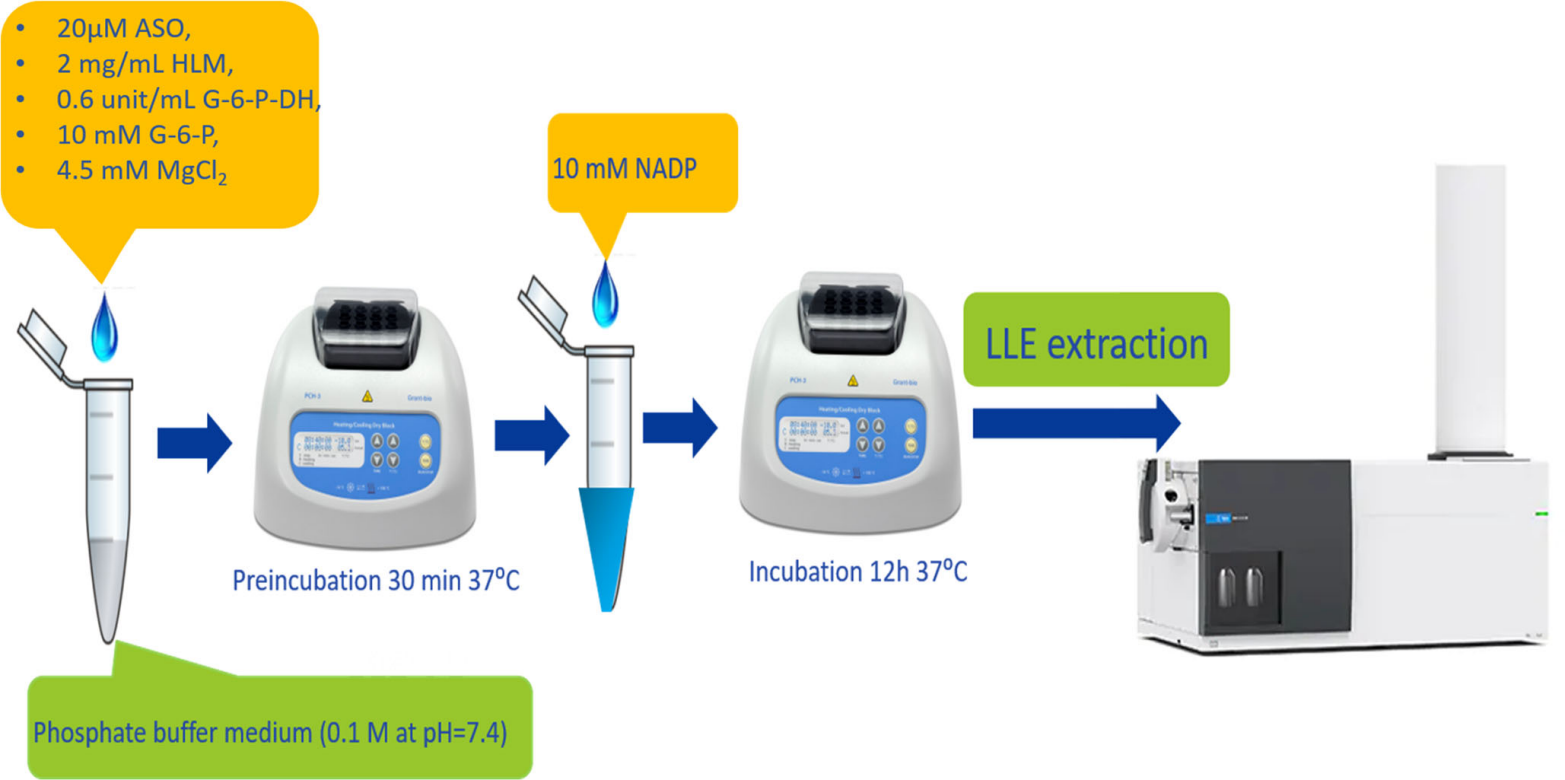
The selected ASO incubation procedure

Based on the results obtained during optimization of incubation conditions, the following parameters were selected: 20 μM of ASO, 2 mg/mL of HLM, 10 mM of NADP, 0.6 U/mL of G-6-P-DH, 10 mM of G-6-P, and 4.5 mM of MgCl_2_. A schematic presentation of the selected incubation procedure is presented in Fig. 3.

After the optimization of incubation conditions as well as the selection of parameters, which provided the greatest number of metabolites, an appropriate in vitro incubation of each ASO with HLM enzymes was performed. The identification of the metabolites was performed with the use of EIC chromatograms and deconvolution tool for the determination of the metabolite mass. Molecular masses of potential metabolites were calculated with the use of the Sigma-Aldrich OligoEvaluatorTM Sequence Analysis (http://www.oligoevaluator.com).

**Fig. 4:**
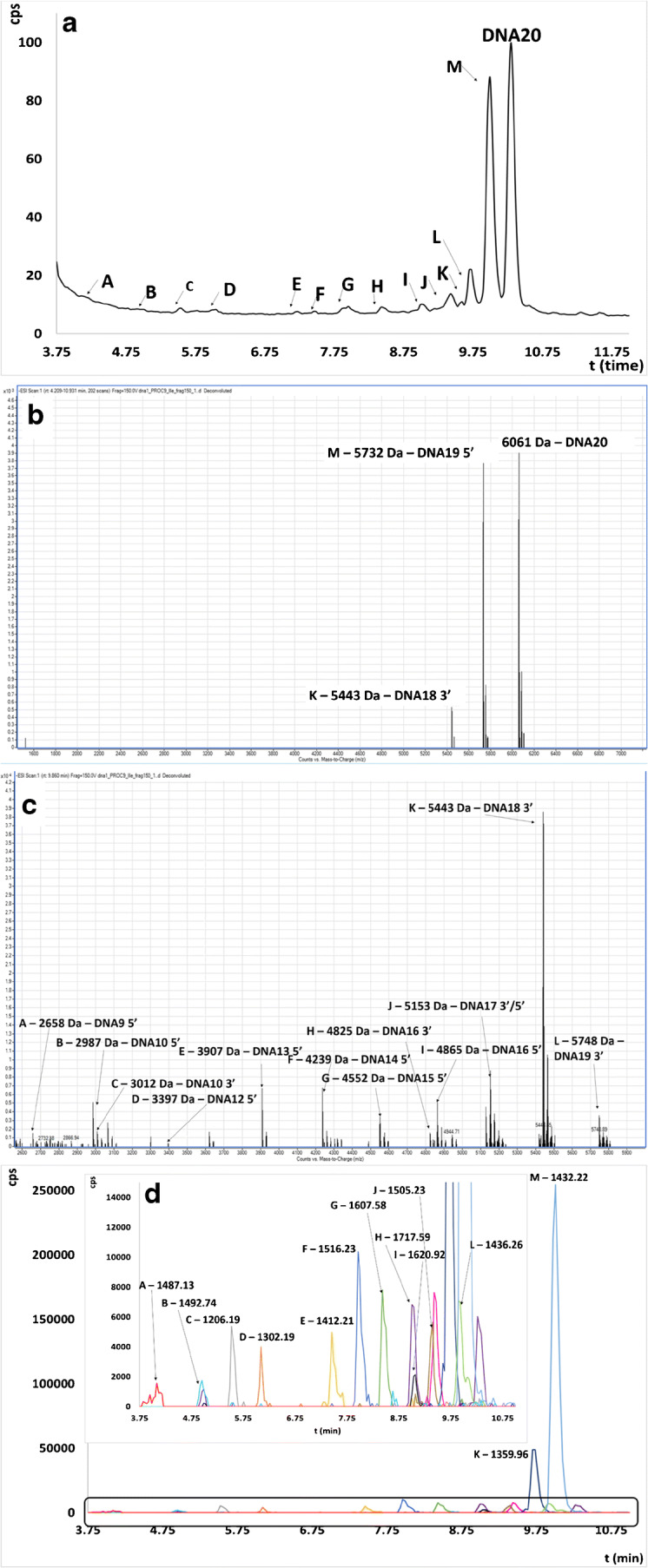
TIC chromatogram (**a**) and deconvoluted full scan spectra for all range of data acquisition (**b**), overlapped deconvoluted full scan spectra for a specific range of data acquisition (**c**), and EIC chromatogram (**d**) obtained for DNA20 after 12 h incubation for procedure 9 (20 μM of ASO, 2 mg/mL of HLM, 10 mM of NADP, 0.6 U/mL of G-6-P-DH, 10 mM of G-6-P, and 4.5 mM of MgCl_2_)

**Fig. 5:**
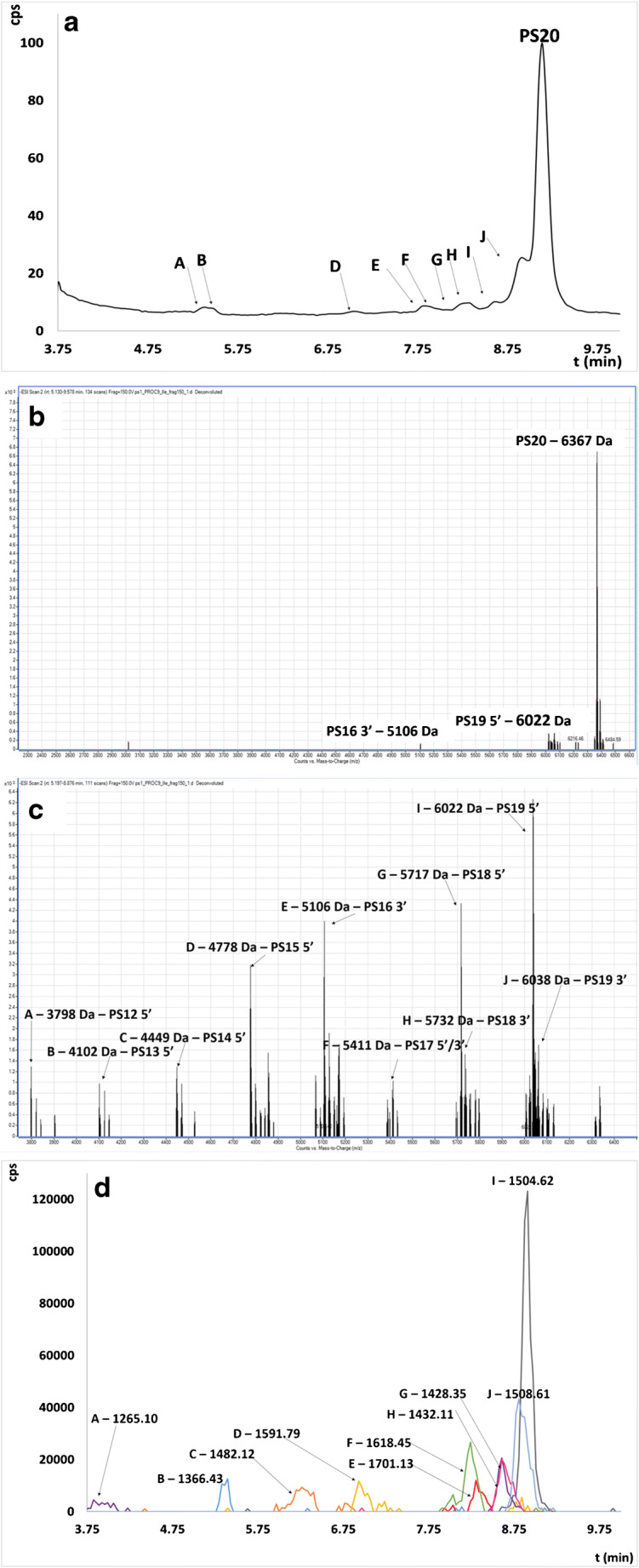
TIC chromatogram (**a**) and deconvoluted full scan spectra for all range of data acquisition (**b**), overlapped deconvoluted full scan spectra for a specific range of data acquisition (**c**), and EIC chromatogram (**d**) obtained for PS20 after 12 h incubation for procedure 9 (20 μM of ASO, 2 mg/mL of HLM, 10 mM of NADP, 0.6 U/mL of G-6-P-DH, 10 mM of G-6-P, and 4.5 mM of MgCl_2_)

Figures 4 and 5 and Figs. S8–S10 in the ESM present TIC, EIC chromatograms, and deconvoluted spectra obtained for each tested ASO.

It may be noted that the greatest number of metabolites was observed in the case of unmodified DNA20 (Fig. 4). However, the biggest EIC peak area was noted for N-1 metabolite from 5′ end as well as N-2 metabolite from 3′ end (Fig.4d). Based on these results, it may be concluded that enzymatic cleavage at the 5′ end of ASO is a predominant metabolism pathway for the unmodified oligonucleotide. Several metabolites were also observed for PS20 (Fig. 5); nonetheless, based on the EIC peak areas of these compounds, it is probable that their amount was significantly lower than in the case of DNA20 (Fig. 5d). This indicates a higher resistance to enzymatic digestion when chemical modifications were introduced to the oligonucleotide structure. The significant number of previous reports demonstrated that the main metabolic pathway of phosphorothioates was based mainly on the 3′-exonuclease activity [11, 12, 15, 16, 40]. Interestingly, our results indicated that their biotransformation is mediated by both 3′- and 5′-exonucleases. Similar tendencies as in the case of PS20 were observed for LNA11, where seven metabolites were identified via deconvolution (ESM Fig. S8). They are formed by enzymatic digestion of ASO at 3′ and 5′ ends. However, a significant ionization suppression was observed in the case of this ASO, which resulted in low signal intensity at full scan spectrum as well as after deconvolution (ESM Fig. S8C). Moreover, a significant number of sodium adducts were observed in the deconvoluted full scan spectrum. Considering TIC chromatogram, it may be noted that there are several undefined peaks for which deconvolution of the full scan spectrum was not useful for their identification. Masses after deconvolution did not indicate metabolites which may be formed by endo- and exonuclease cleavage. For this reason, in vitro metabolism studies for this ASO should be further continued. A greater number of metabolites for LNA11 were identified compared to ME20 and MOE20, probably due to the shorter sequence of this oligonucleotide. Only four metabolites shorter by one and two nucleotides from 5′ and 3′ ends were observed for ME20 (ESM Fig. S9). Moreover, the signal of 6300 Da has been identified as 3′N-1 with sodium adduct. As has been mentioned above MOE20 was highly resistant for enzymatic digestion and only one metabolite was observed, namely N-1 shorter metabolism product at 3′ end with molecular mass 7268 Da (ESM Fig. S10). Modification in the ribose structure of ASOs also significantly increases the plasma half-lives, and consequently, these compounds are metabolized more slowly [9, 28, 35, 38, 41]. For this reason, further optimization of incubation time should be considered for these ASOs. Another reason of the lower number of detected metabolites for ME and MOE may be not only related to their higher resistance against nucleases and too short incubation time but also with the ionization effectiveness of these compounds related to their resultant hydrophobicity. As can be seen in Table 3, there was a significant decrease in the EIC peak areas for these modifications, which also may influence the number of detected metabolites.

## Conclusions

In summary, it may be concluded that IPC provided greater sensitivity of oligonucleotide analysis compared to HILIC mode. Such an effect was probably a consequence of the presence of aprotic acetonitrile in the mobile phase as well as ammonium formate. Consequently, greater signal suppression occurred, compared to the IPC mode. IPC UHPLC provided selective separation of all tested ASO mixtures in short analysis time. Optimization of incubation parameters was crucial because changing the concentration of each component of reaction buffer significantly influenced the number of metabolites obtained for each tested ASO. Moreover, the application of the LLE with the use of phenol/chloroform/isoamyl alcohol allowed us to remove matrix interferences as well as purification of the samples. Studying of in vitro metabolism of ASOs with the use of IPC-Q-TOF-MS showed that the main metabolism pathway of these compounds includes the enzymatic cleavage by intracellular nucleases at both 3′ and 5′ ends of the oligonucleotide. Such an effect was observed regardless of the ASO modification as well as sequence length. Moreover, it may be noted that increasing the hydrophobicity of modification in the ASO structure increases its resistance to enzymatic digestion. It needs to be highlighted that in vitro studies with human liver microsomes of such a wide range of the different oligonucleotides was performed for the first time. For this reason, we can conclude that this in vitro model is useful for the investigation of the biotransformation of the various ASO modifications.

## Electronic supplementary material

ESM 1 (PDF 2486 kb)
