# Supplementary material for: Studying in vitro metabolism of the first and second generation of antisense oligonucleotides with the use of ultra-high-performance liquid chromatography coupled with quadrupole time-of-flight mass spectrometry
Source: Anal Bioanal Chem. 2020 Aug 27;412(27):7453–67. doi: 10.1007/s00216-020-02878-0 (PMC7533254; doi:10.1007/s00216-020-02878-0)
Supplement: Supplementary file 1 — (PDF 2486 kb) [file 216_2020_2878_MOESM1_ESM.pdf]

Analytical and Bioanalytical Chemistry

Electronic Supplementary Material

Studying in vitro metabolism of the first and second generation of antisense oligonucleotides with the use of ultra high performance liquid chromatography coupled with quadrupole time-of-flight mass spectrometry

Anna Kilanowska, Łukasz Nuckowski, Sylwia Studzińska

Table S1 Optimized gradient elution programs and mobile phase flow rates used during separation of model ASOs mixtures and ASOs analysis after incubation

Tested ASO mixture	Mixture composition	Modification	Gradient elution program developed for model ASOs mixtures separation	Mobile phase flow rate for model ASOs mixtures separation (mL/min)	Gradient elution programs developed for ASOs samples after incubation	Mobile phase flow rate for ASOs samples after incubation (mL/min)
MIX1	DNA20, DNA19, DNA18	unmodified	16-25% v/v MeOH in 10 min.	0.35	16-30% v/v MeOH in 10 min.	0.32
MIX2	PS20, PS19, PS18	phosphorothioate	20-25% v/v MeOH in 10 min.	0.32	20-35% v/v MeOH in 10 min.	0.32
MIX3	ME20, ME19, ME18	2'-O-methyl	21-25% v/v MeOH in 10 min.	0.35	21-35% v/v MeOH in 10 min.	0.32
MIX4	MOE20, MOE19, MOE18	2'-O-methoxyethyl	30-36% v/v MeOH in 10 min.	0.325	30-45% v/v MeOH in 10 min.	0.3
MIX5	LNA11, LNA10, LNA9	Methylene bridge between 2'-O and 4'-C	10-23% v/v MeOH in 10 min.	0.35	10-30% v/v MeOH in 10 min.	0.32

Table S2 Fully coded CCD and responses obtained for DNA20 for three different sets of variables

**DGT (^oC)**	**SGT (□C)**	**CV (V)**	**TIC peak area for IPC mode**	**TIC peak area for HILIC mode**
50	50	3700	9163254	3705950
50	50	600	0	0
50	250	3700	2221135	18978808
190	43	3500	0	0
230	50	600	0	0
40	210	3500	25200448	17476431
50	250	600	0	0
230	50	3700	20333249	12729998
190	210	3500	49334463	39342072
190	210	6000	0	0
190	210	900	13005946	6264405
230	250	3700	28658535	26194316
190	380	3500	25711927	24975042
340	210	3500	33239514	28164777
230	250	600	0	0
**Skimmer (V)**	**Octopole (V)**	**Fragmentor (V)**	**TIC peak area**	**TIC peak area**
160	410	170	48804032	3636365
300	410	170	0	0
160	17	170	0	0
190	20	200	0	0
160	410	20	0	0
20	490	200	172696283	12995909
190	20	20	0	0
190	490	20	0	0
18	410	170	68439737	12581864
160	800	170	50851673	11467329
190	490	200	63817817	7981938.5
20	490	20	13957435	5167908
20	20	20	0	0
160	410	320	150278506	9134948
20	20	200	0	8745140.5
**DGF (L/min)**	**SGF (L/min)**	**Nebulizer (psig)**	**TIC peak area**	**TIC peak area**
6	10	30	70873175	3097520.5
6	8	30	69295737.5	3943929
1.8	10	30	50935587.5	3736467
8	11	40	45369945	4151109.5
3	11	40	32130784	5068277
8	11	15	73559514.5	6932277.5
8	9	15	105005813	6911889.5
3	11	15	84688123	10078308
6	10	50	41566646.5	5008441

3	9	40	44393001.5	2481566
6	10	9	66544487	6641073.5
6	11.6	30	63896500	6647063.5
10	10	30	76545273.5	2118680.5
8	9	40	363668221	5166393
3	9	15	89448672	5868235

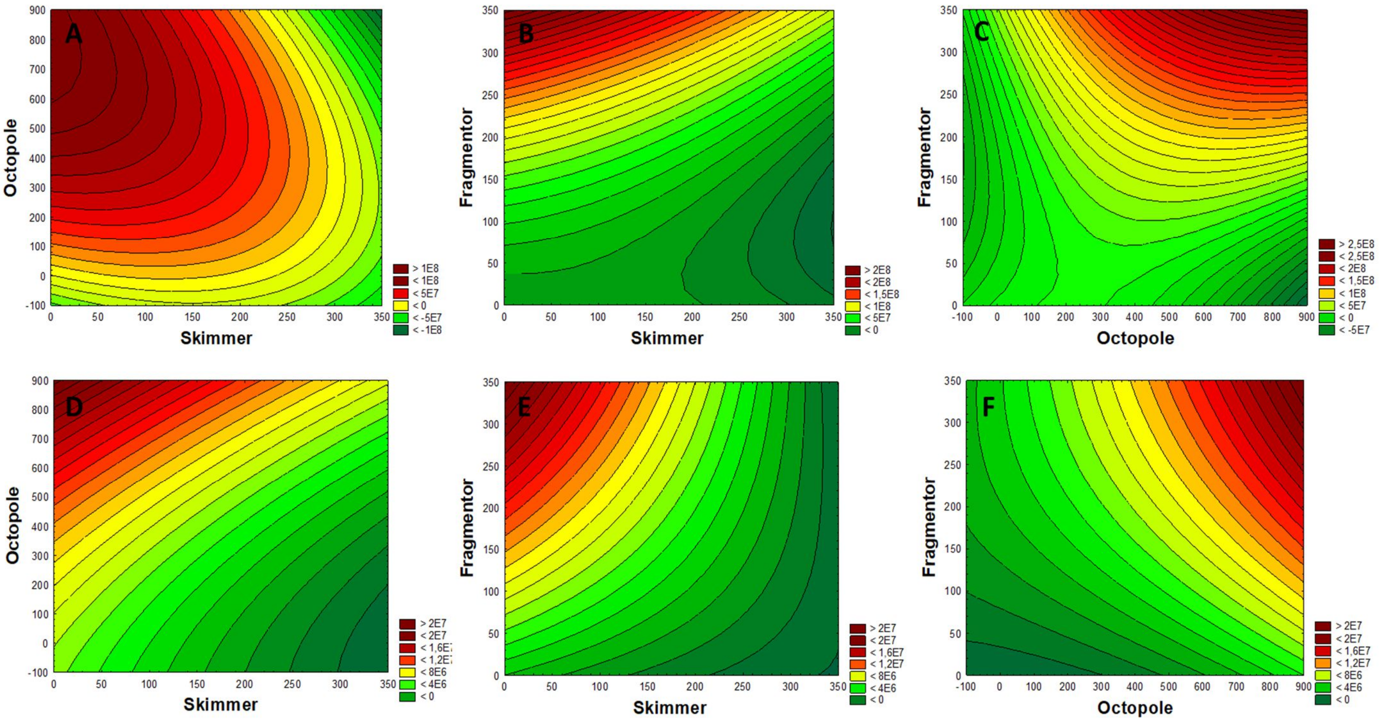

Fig. S1 Exemplary 2D contour plots obtained plotting CCD equation for Q-TOF-MS optimization for DNA20 in IPC (A-C) and HILIC (D-F) mode A) octopole vs. skimmer is plotted, B) fragmentor vs. skimmer is plotted, C) fragmentor vs. octopole is plotted, D) octopole vs. skimmer is plotted, E) CV vs. DGT is plotted, F) CV vs. SGT is plotted. For each graph the third variable is TIC peak area

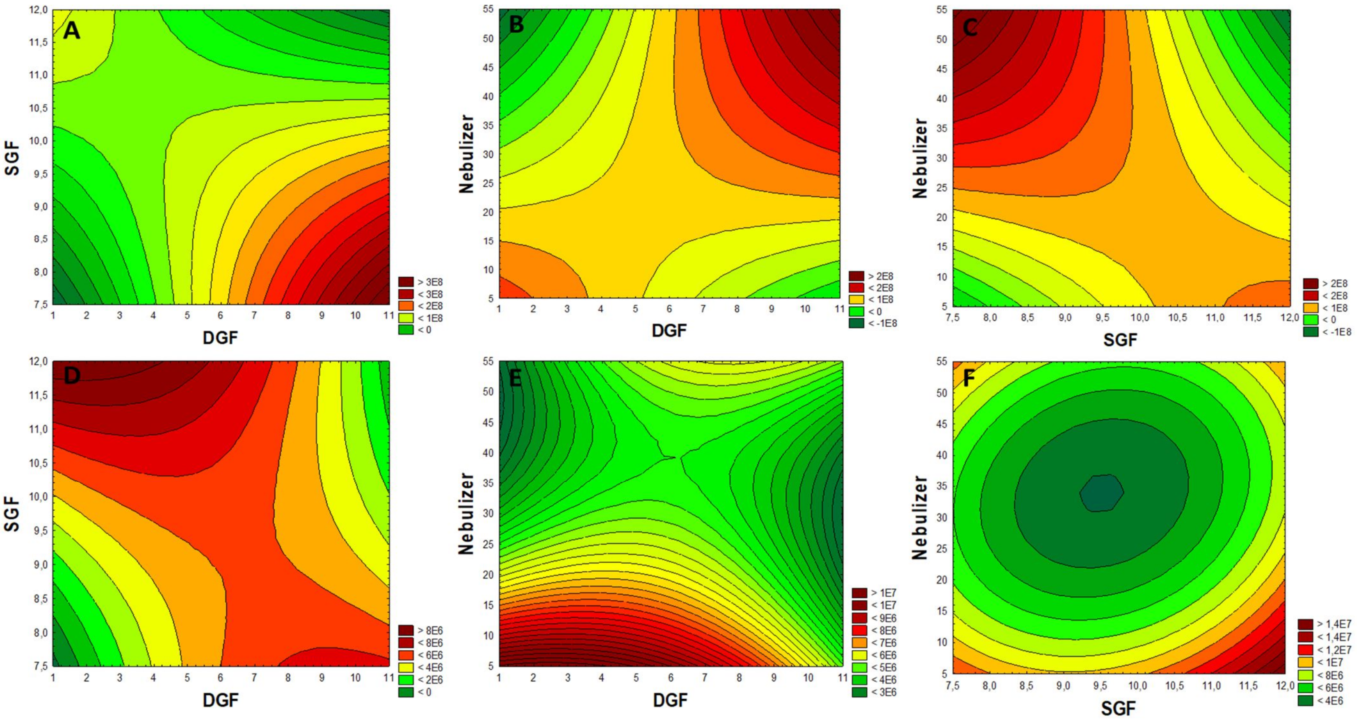

Fig. S2 Exemplary 2D contour plots obtained plotting CCD equation for Q-TOF-MS optimization for DNA20 in IPC (A-C) and HILIC (D-F) mode A) SGF vs. DGF is plotted, B) Nebulizer vs. DGF is plotted, C) Nebulizer vs. SGF is plotted, D) SGF vs. DGF is plotted, E) Nebulizer vs. DGF is plotted, F) Nebulizer vs. SGF is plotted. For each graph the third variable is TIC peak area

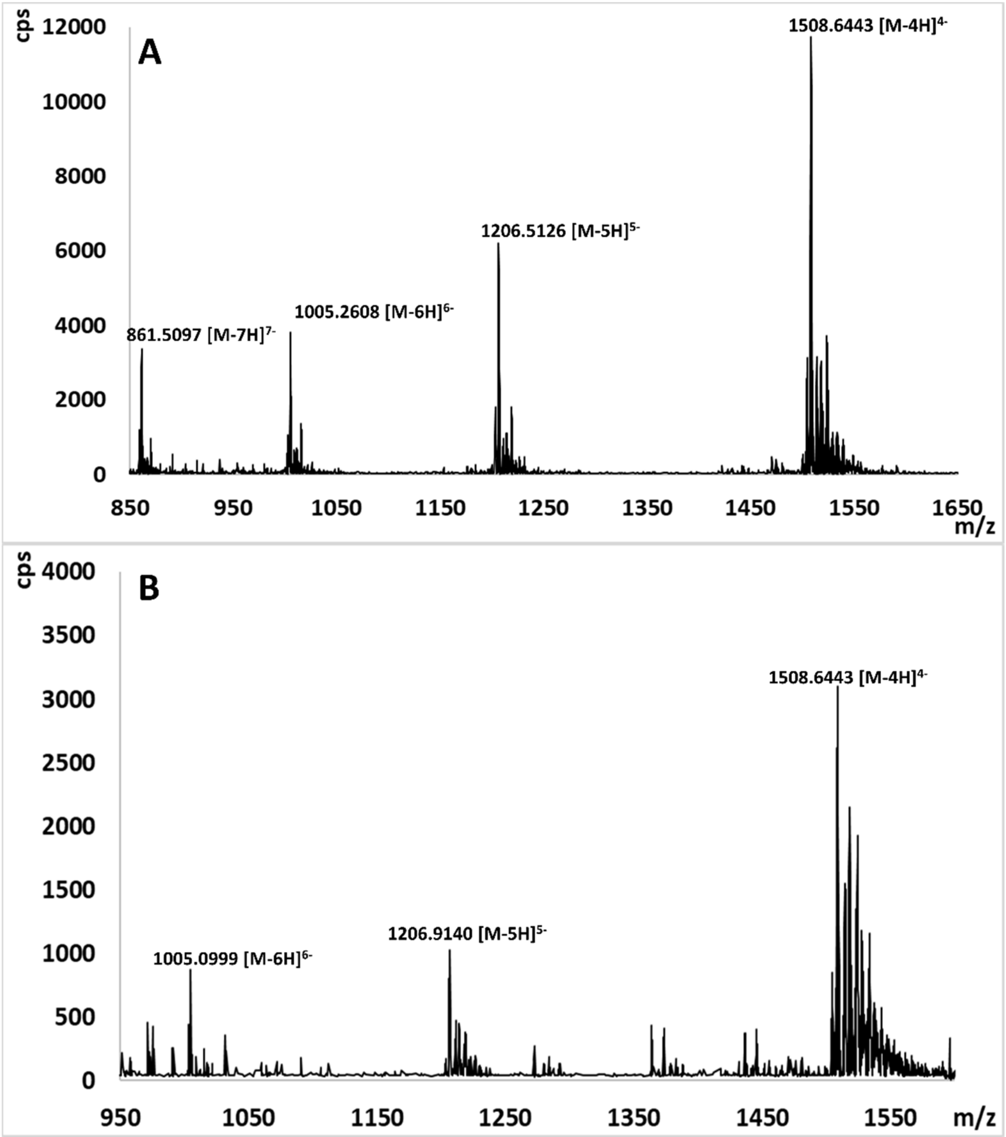

Fig. S3 Exemplary FS spectra obtained for PS19 A) in IPC mode; B) in HILIC mode. Chromatographic conditions and MS parameters: A) 80% v/v 5 mM DMBA/ 150 mM HFIP and 20% v/v MeOH, SGT - 350°C, DGT - 400°C, CV - 3500 V, octopole - 800 V, fragmentor - 150 V, skimmer 60 V, SGF - 8 L/min, DGF - 11 L/min, and nebulizer pressure - 50 psig B) 30% v/v 5 mM pH 7.5 ammonium formate and 70% v/v acetonitrile, SGT - 350°C, DGT - 400 °C, CV - 4000 V, octopole - 800 V, fragmentor - 150 V and skimmer - 60 V, SGF - 12 L/min, DGF - 3 L/min, nebulizer pressure - 15 psig. Injection volume – 0.5 µL of 25 µM ASO

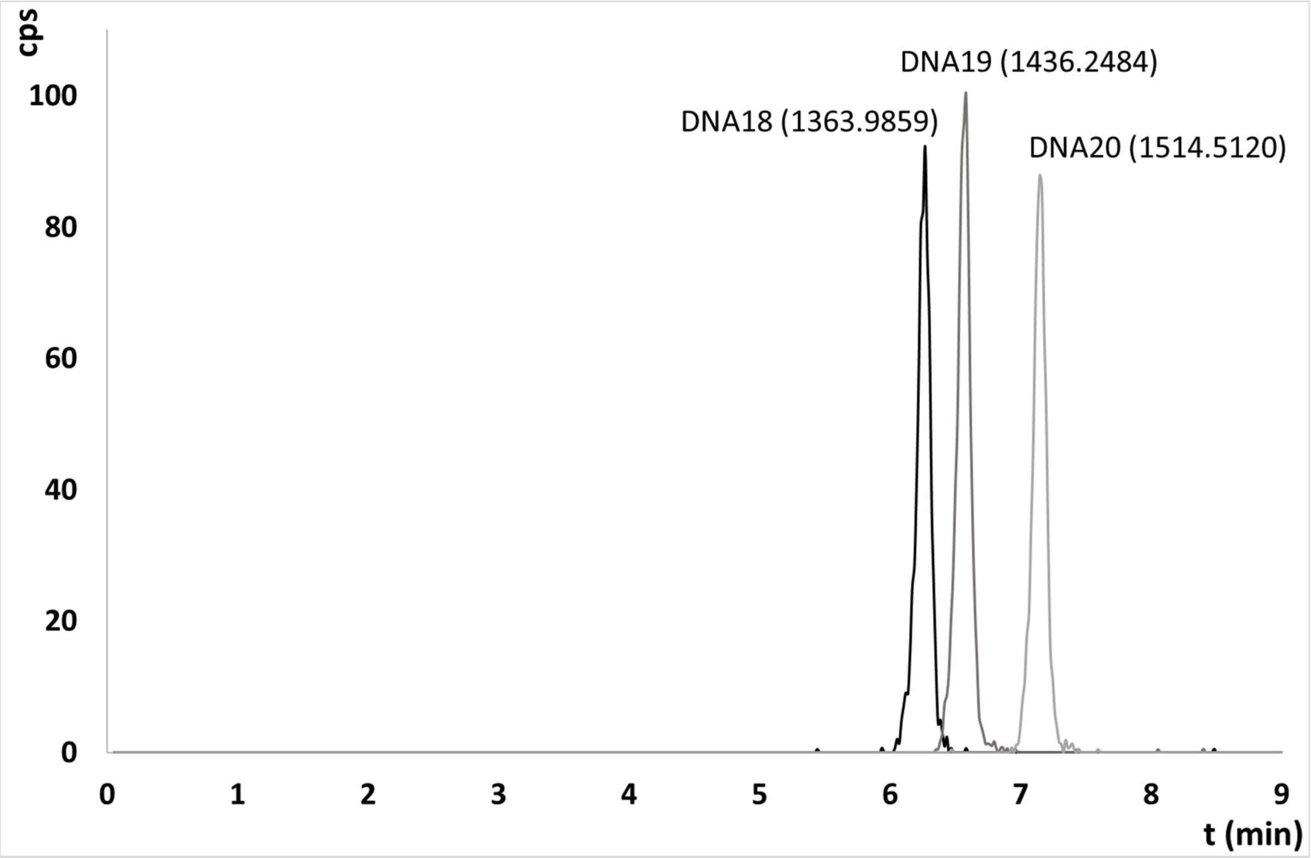

Fig. S4 Exemplary EIC chromatogram obtained for DNA20, DNA19 and DNA18 mixture. Chromatographic conditions: see section “Materials and methods” and Table S1

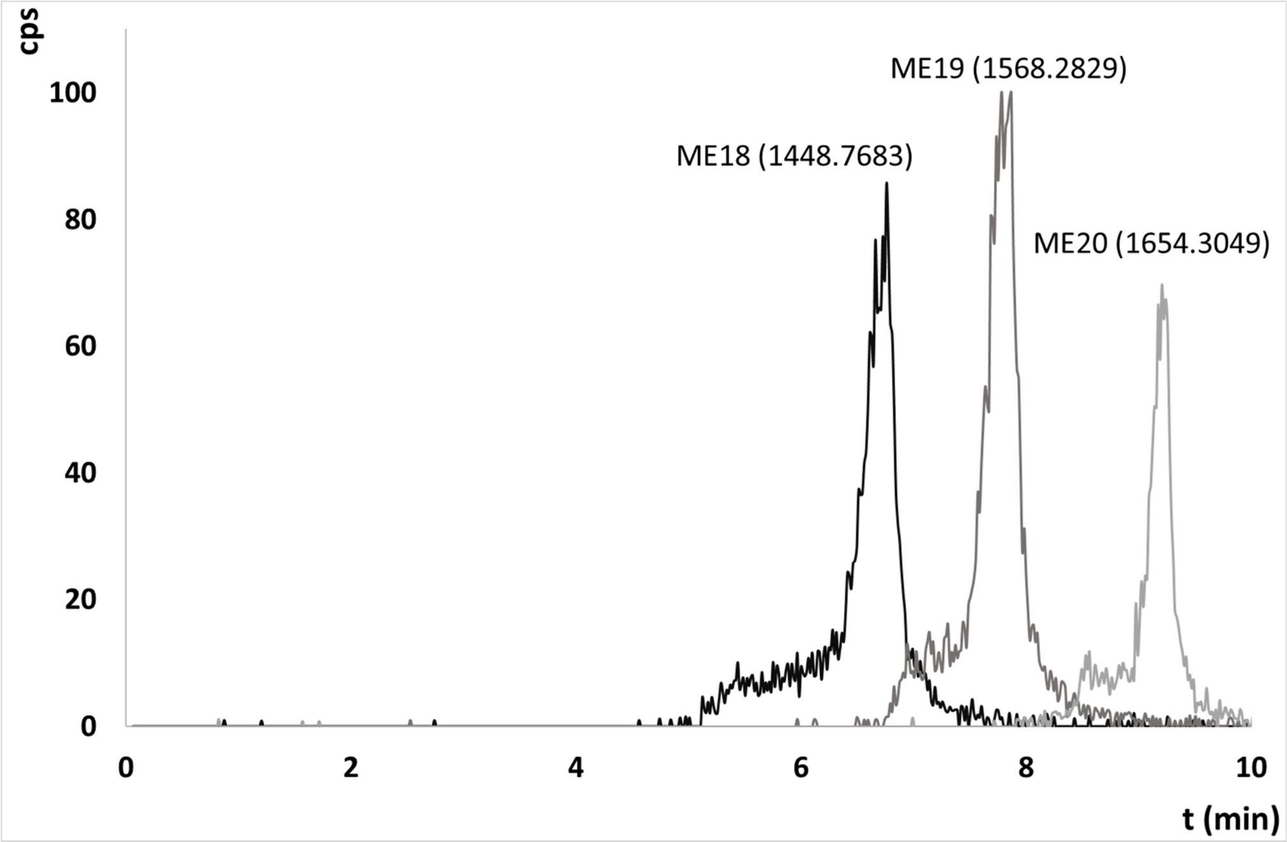

Fig. S5 Exemplary EIC chromatogram obtained for ME20, ME19 and ME18 mixture. Chromatographic conditions: see section "Materials and methods" and Table S1

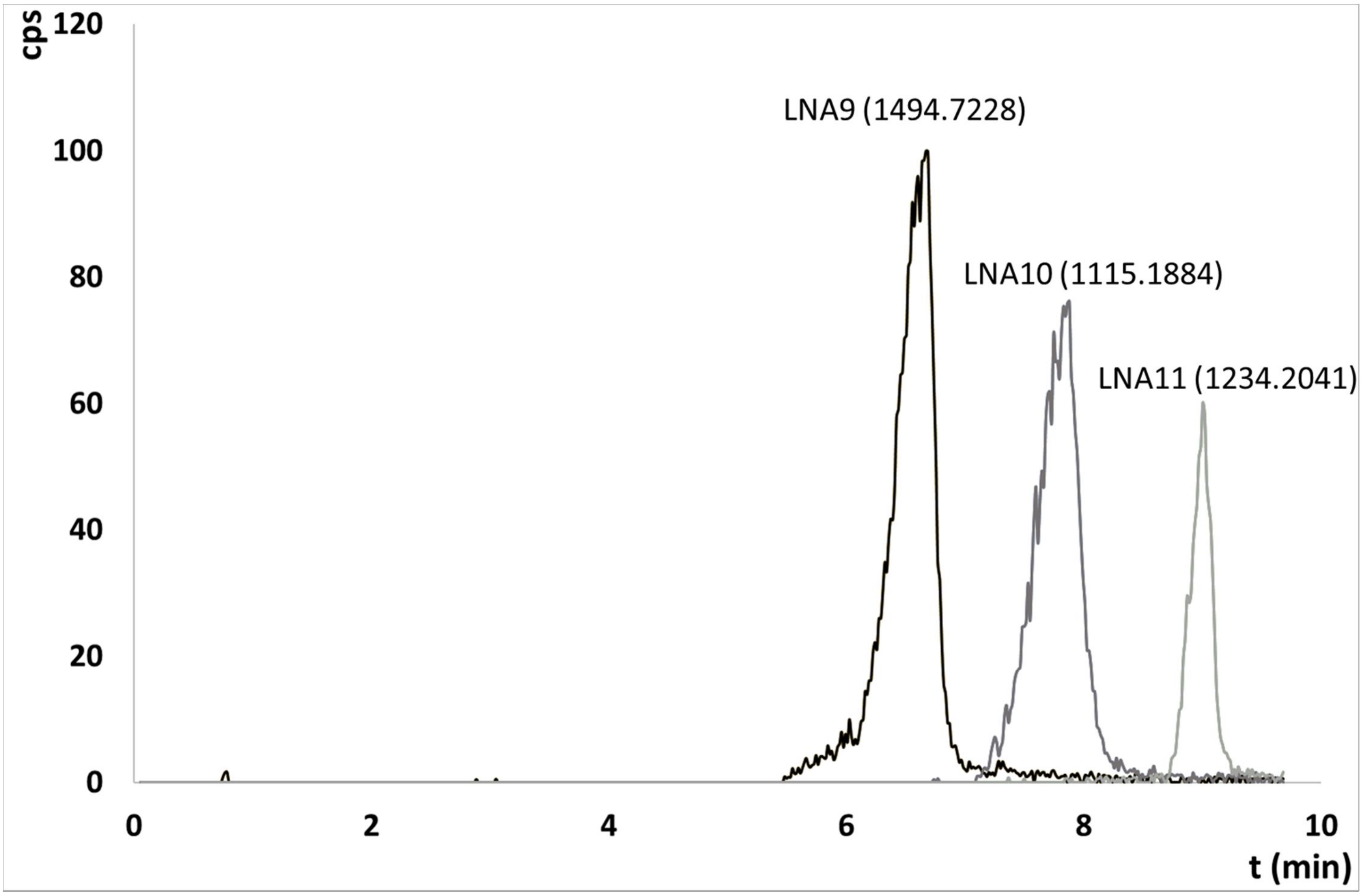

Fig. S6 Exemplary EIC chromatogram obtained for LNA11, LNA10 and LNA9 mixture. Chromatographic conditions: see section “Materials and methods” and Table S1

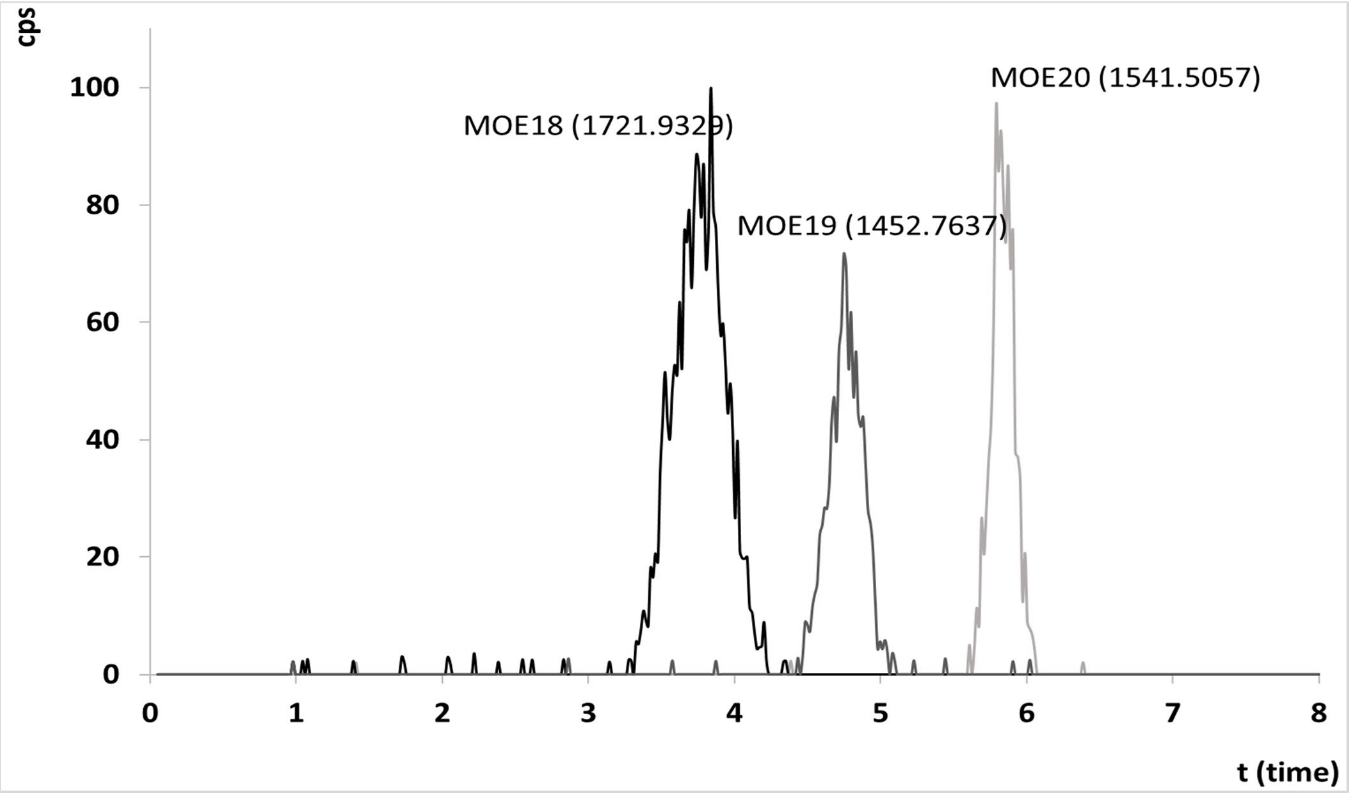

Fig. S7 Exemplary EIC chromatogram obtained for MOE20, MOE19 and MOE18 mixture. Chromatographic conditions: see section “Materials and methods” and Table S1

Table S3 In vitro-generated ASOs metabolites after incubation with human liver homogenates for different incubation procedures

Metabolite	Molecular Mass (Da)	P1	P2	P3	P4	P5	P6	P7	P8	P9	P10	P11	P12	P13	P14	P15
**DNA20**																
**3'N-1**	**5748**	+	+	+	+	+	+	n.a.	+	+	-	+	+	+	+	+
**5'N-1**	**5732**	+	+	+	+	+	+	n.a.	+	+	+	+	+	+	+	+
**3'N-2**	**5458**	+	+	+	-	-	-	n.a.	-	-	-	-	+	+	-	-
**5'N-2**	**5443**	-	-	-	+	+	+	n.a.	+	+	+	+	+	+	+	+
**3'N-3**	**5154**	+	+	+	+	+	+	n.a.	+	+	+	+	+	+	+	+
**5'N-3**	**5154**	+	+	+	+	+	+	n.a.	+	+	+	+	+	+	+	+
**3'N-4**	**4827**	-	-	-	-	+	+	n.a.	+	+	+	-	+	+	+	+
**5'N-4**	**4865**	-	-	+	+	+	-	n.a.	+	+	+	+	+	-	+	+
**5'N-5**	**4552**	-	-	-	-	-	+	n.a.	+	+	+	+	+	+	+	+
**5'N-6**	**4238**	-	-	-	-	-	-	n.a.	+	+	+	+	+	+	+	+
**5'N-7**	**3910**	-	-	-	-	+	+	n.a.	+	+	+	+	+	-	+	+
**5'N-8**	**3621**	-	-	-	-	-	-	n.a.	+	+	+	+	-	-	-	-
**5'N-9**	**3317**	-	-	-	-	-	-	n.a.	-	+	+	+	-	-	-	-
**3'N-10**	**3011**	-	-	-	-	-	+	n.a.	+	+	-	+	-	-	+	-
**5'N-10**	**2987**	-	-	-	-	-	-	n.a.	-	+	-	+	+	+	+	+
**3'N-11**	**2680**	-	-	-	-	-	-	n.a.	+	+	-	-	-	-	+	-
**5'N-11**	**2658**	-	-	-	-	-	-	n.a.	+	-	-	-	+	+	+	-
**PS20**																
**3'N-1**	**6038**	-	-	-	-	+	+	+	+	+	+	+	+	-	+	+
**5'N-1**	**6022**	-	+	+	+	+	+	+	+	+	+	+	+	-	+	+
**3'N-2**	**5732**	-	+	+	-	-	-	-	-	+	-	-	-	-	-	-
**5'N-2**	**5716**	-	-	+	-	-	+	-	+	+	-	-	-	-	-	-
**3'N-3**	**5413**	-	-	-	-	-	-	-	+	+	-	-	-	-	-	-
**5'N-3**	**5413**	-	-	-	-	-	-	-	+	+	-	-	-	-	-	-
**3'N-4**	**5067**	-	-	-	+	-	-	-	+	+	-	-	-	-	-	-
**5'N-4**	**5105**	-	-	-	+	-	-	-	+	+	-	-	+	-	-	-
**3'N-5**	**4761**	-	-	-	-	-	-	-	+	+	-	-	-	-	+	-
**5'N-5**	**4777**	-	-	-	+	-	-	+	+	+	+	-	-	-	-	+
**5'N-6**	**4448**	-	-	-	-	-	-	+	+	+	-	-	-	-	-	-
**5'N-7**	**4102**	-	-	-	+	-	-	-	+	+	+	+	-	-	-	-
**5'N-8**	**3798**	-	-	-	-	-	-	-	+	+	-	-	-	-	-	-

**ME20**																
**3’N-1**	**6278**	-	-	-	-	-	-	+	+	+	-	+	-	-	+	-
**3’N-1+sodium adduct**	**6301**	-	-	-	+	+	+	+	+	+	+	+	+	-	+	+
**5’N-1**	**6261**	-	-	-	+	-	+	+	+	+	+	+	-	-	+	+
**3’N-2**	**5957**	-	-	-	-	-	+	+	+	+	-	+	-	-	-	-
**5’N-2**	**5941**	-	-	-	-	+	+	-	+	+	-	+	-	-	-	-
**MOE20**																
**3’N-1**	**7268**	-	-	-	-	-	-	-	-	+	-	-	-	-	-	-
**LNA11**																
**3’N-1**	**3348**	-	+	+	+	+	+	n.a.	+	+	+	+	-	-	+	+
**5’N-1**	**3348**	-	+	+	+	+	+	n.a.	+	+	+	+	-	-	+	+
**3’N-2**	**2990**	-	-	-	+	+	+	n.a.	+	+	+	+	+	+	+	+
**5’N-2**	**3016**	-	-	-	-	-	-	n.a.	-	-	-	+	-	-	+	+
**Hydrated 5’N-2 without thymine**	**2847**	-	+	+	+	+	+	n.a.	+	+	-	-	+	-	-	-
**3’N-3**	**2659**	-	-	+	-	+	+	n.a.	+	+	+	+	+	+	+	+
**5’N-3**	**2684**	-	-	-	+	-	-	n.a.	+	+	+	+	-	-	-	+
**3’N-4**	**2328**	-	-	-	-	+	+	n.a.	+	+	+	-	-	-	+	+
**5’N-4**	**2354**	-	-	-	-	+	+	n.a.	-	-	-	+	-	-	-	-
**3’N-5**	**1970**	-	-	-	-	+	+	n.a.	+	+	+	+	+	-	+	+

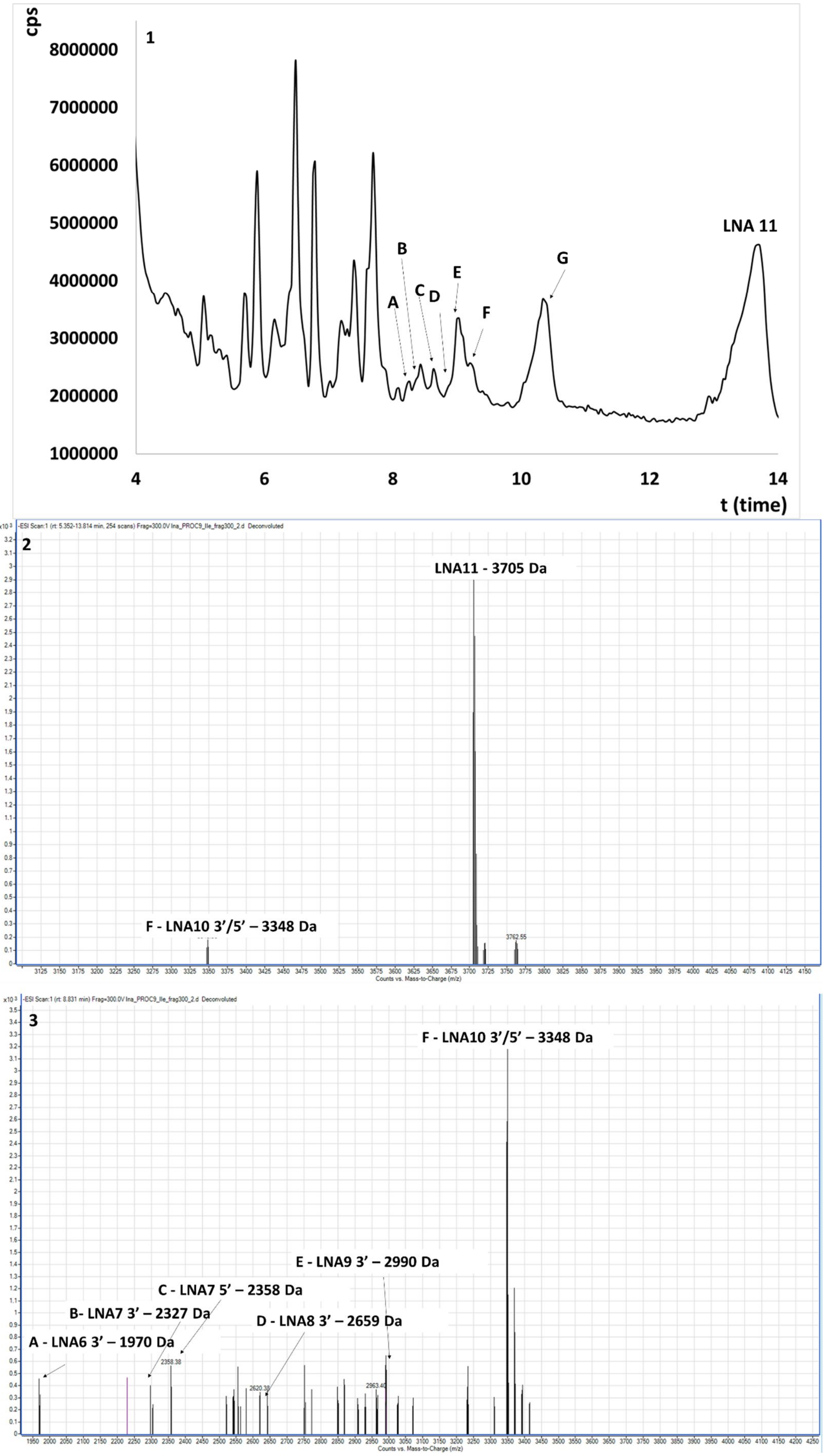
cps
1
8000000
7000000
6000000
5000000
4000000
3000000
2000000
1000000
4
6
8
10
12
14
t (time)
A
B
C
D
E
F
G
LNA 11
-ESI Scan:1 (rt: 5.352-13.814 min, 254 scans) Frag=300.0V lna_PROC9_lle_frag300_2.d Deconvoluted
2
LNA11 - 3705 Da
F - LNA10 3'/5' – 3348 Da
3762.55
Counts vs. Mass-to-Charge (m/z)
-ESI Scan:1 (rt: 8.831 min) Frag=300.0V lna_PROC9_lle_frag300_2.d Deconvoluted
3
F - LNA10 3'/5' – 3348 Da
E - LNA9 3' – 2990 Da
C - LNA7 5' – 2358 Da
B- LNA7 3' – 2327 Da
D - LNA8 3' – 2659 Da
A - LNA6 3' – 1970 Da
2358.38
2620.38
2963.40
Counts vs. Mass-to-Charge (m/z)

Fig. S8 TIC chromatogram (1), and deconvoluted full scan spectra for the all range of data acquisition (2), overlapped deconvoluted full scan spectra for a specific ranges of data acquisition (3) obtained for LNA11 after 12h incubation for Procedure 9 (20 µM of ASO, 2 mg/ml of HLM, 10 mM of NADP, 0.6 uN/ml of G-6-P-DH, 10 mM of G-6-P and 4.5mM of $MgCl_2$)

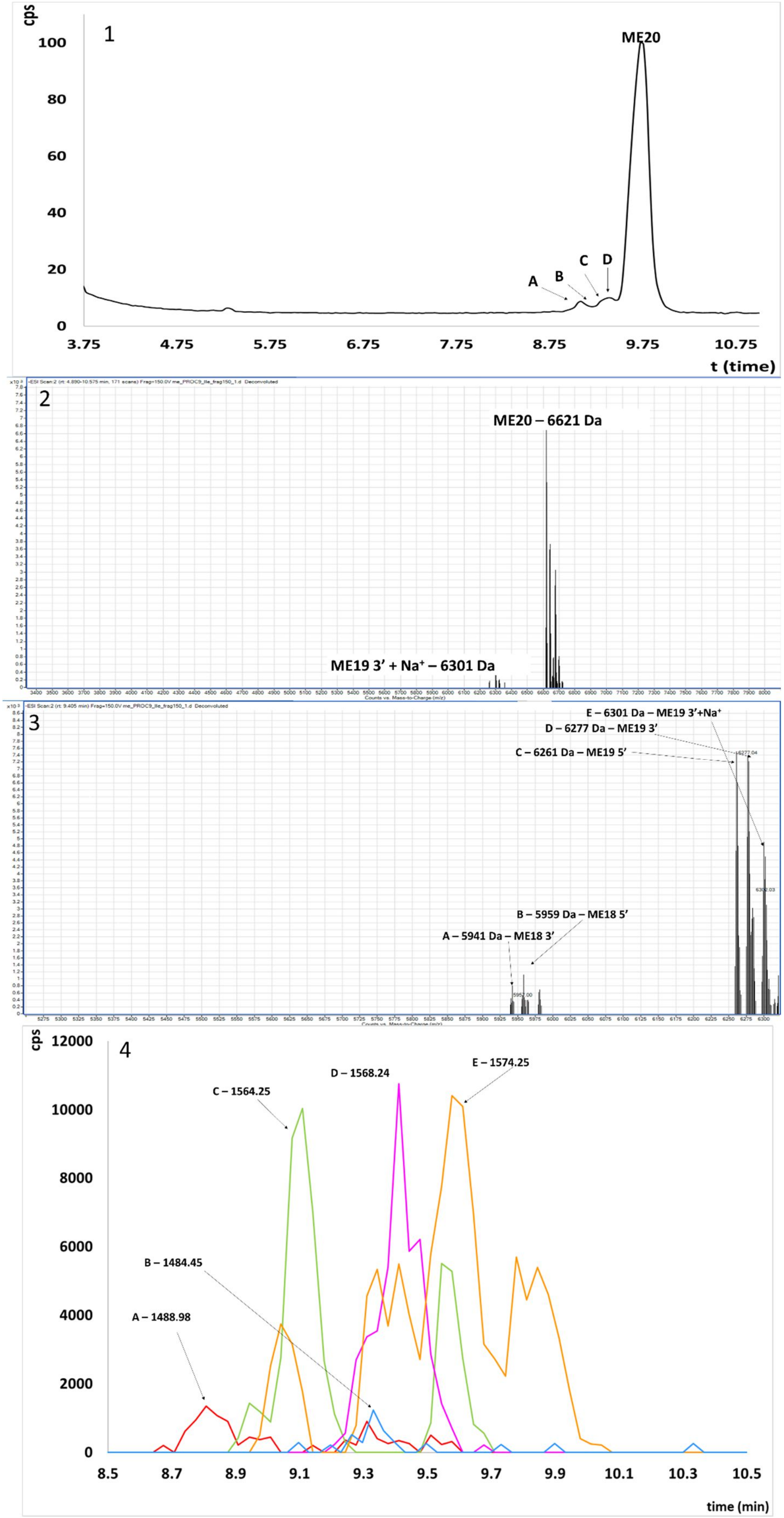
cps
1
ME20
A
B
C
D
t (time)
2
ME20 – 6621 Da
ME19 3’ + Na⁺ – 6301 Da
3
E – 6301 Da – ME19 3’+Na⁺
D – 6277 Da – ME19 3’
C – 6261 Da – ME19 5’
B – 5959 Da – ME18 5’
A – 5941 Da – ME18 3’
4
C – 1564.25
D – 1568.24
E – 1574.25
B – 1484.45
A – 1488.98
time (min)

Fig. S9 TIC chromatogram (1), and deconvoluted full scan spectra for the all range of data acquisition (2), overlapped deconvoluted full scan spectra for a specific ranges of data acquisition (3) and EIC chromatogram (4) obtained for ME20 after 12h incubation for Procedure 9 (20 μM of ASO, 2 mg/ml of HLM, 10 mM of NADP, 0.6 uN/ml of G-6-P-DH, 10 mM of G-6-P and 4.5mM of $MgCl_2$)

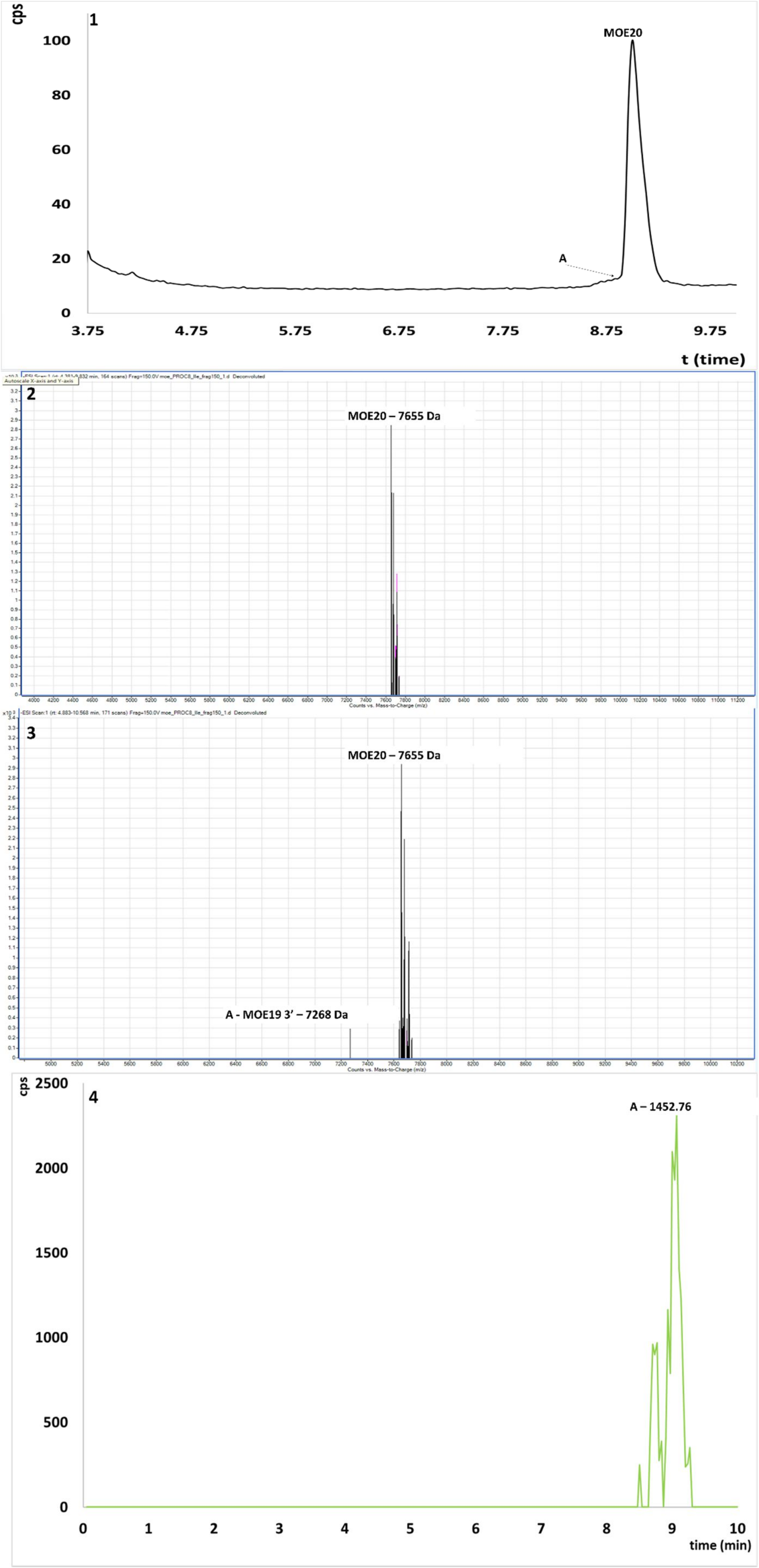
1
cps
100
80
60
40
20
0
3.75
4.75
5.75
6.75
7.75
8.75
9.75
t (time)
MOE20
A
2
MOE20 – 7655 Da
Counts vs. Mass-to-Charge (m/z)
3
MOE20 – 7655 Da
A - MOE19 3' – 7268 Da
Counts vs. Mass-to-Charge (m/z)
4
cps
2500
2000
1500
1000
500
0
0
1
2
3
4
5
6
7
8
9
10
time (min)
A – 1452.76

Fig. S10 TIC chromatogram (1), and deconvoluted full scan spectra for the all range of data acquisition (2), overlapped deconvoluted full scan spectra for a specific ranges of data acquisition (3) and EIC chromatogram obtained for MOE20 (4) after 12h incubation for Procedure 9 (20 μM of ASO, 2 mg/ml of HLM, 10 mM of NADP, 0.6 uN/ml of G-6-P-DH, 10 mM of G-6-P and 4.5mM of $MgCl_2$)
